# Marine heatwaves modulate the genotypic and physiological responses of reef‐building corals to subsequent heat stress

**DOI:** 10.1002/ece3.10798

**Published:** 2023-12-13

**Authors:** Kristen T. Brown, Amatzia Genin, Matheus A. Mello‐Athayde, Ellie Bergstrom, Adriana Campili, Aaron Chai, Sophie G. Dove, Maureen Ho, Devin Rowell, Eugenia M. Sampayo, Veronica Z. Radice

**Affiliations:** ^1^ School of Biological Sciences University of Queensland St Lucia Queensland Australia; ^2^ Department of Biology University of Pennsylvania Philadelphia Pennsylvania USA; ^3^ The Interuniversity Institute for Marine Sciences of Eilat The Hebrew University of Jerusalem Eilat Israel; ^4^ CarbonDrop San Carlos California USA; ^5^ Australian Institute of Marine Science Townsville Mail Centre Townsville Queensland Australia; ^6^ Faculty of Science and Engineering Southern Cross University East Lismore New South Wales Australia; ^7^ Cawthron Institute Nelson New Zealand; ^8^ Department of Biological Sciences Old Dominion University Norfolk Virginia USA

**Keywords:** climate change, coral bleaching, corals reefs, ecological memory, heat stress, legacy effects, thermal history, trophic plasticity

## Abstract

Back‐to‐back marine heatwaves in 2016 and 2017 resulted in severe coral bleaching and mortality across the Great Barrier Reef (GBR). Encouragingly, some corals that survived these events exhibit increased bleaching resistance and may represent thermally tolerant populations that can better cope with ocean warming. Using the GBR as a natural laboratory, we investigated whether a history of minimal (Heron Island) or severe (Lizard Island) coral bleaching in 2016 and 2017 equates to stress tolerance in a successive heatwave (2020). We examined the genetic diversity, physiological performance, and trophic plasticity of juvenile (<10 cm) and adult (>25 cm) corals of two common genera (*Pocillopora* and *Stylophora*). Despite enduring greater cumulative heat stress (6.3°C week^−1^ vs. 5.6°C week^−1^), corals that experienced the third marine heatwave in 5 years (Lizard) exhibited twice as high survival and visual bleaching thresholds compared to corals that had not experienced significant bleaching in >10 years (Heron). Surprisingly, only one shared host–Symbiodiniaceae association was uncovered between locations (*Stylophora pistillata*–*Cladocopium* “C8 group”) and there was no genetic overlap in *Pocillopora*–*Cladocopium* partnerships, suggesting turnover in species composition from recent marine heatwaves. Corals within the species complex *Pocillopora* that survived the 2016 and 2017 marine heatwaves at Lizard Island were the most resilient, exhibiting three times greater calcification rates than conspecifics at Heron Island. Further, surviving corals (Lizard) had distinct isotopic niches, lower host carbon, and greater host protein, while conspecifics that had not experienced recent bleaching (Heron) had two times greater symbiont carbon content, suggesting divergent trophic strategies that influenced survival (i.e., greater reliance on heterotrophy vs. symbiont autotrophy, respectively). Ultimately, while corals may experience less bleaching and survive repeated thermal stress events, species‐specific trade‐offs do occur, leaving open many questions related to the long‐term health and recovery of coral reef ecosystems in the face of intensifying marine heatwaves.

## Graphical abstract

Coral reefs are in serious danger, with accelerating ocean warming currently considered the greatest threat to reef survival. This study identifies that ecological memory of severe coral bleaching promoted survival and bleaching resistance in a successive heatwave three years later through physiological legacies and shifts in community composition.

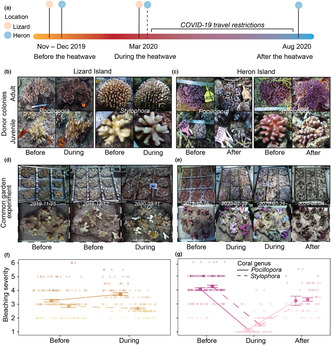

## 1 INTRODUCTION

Coral reefs are among the most threatened ecosystems on Earth, with half of the world's reef‐building corals lost since 1950 (Eddy et al., 2021). As climate change intensifies, marine heatwaves are now occurring on multidecadal time‐scales, and even in back‐to‐back years, leading to repeated and worsening mass coral bleaching events (Gintert et al., 2018; Hughes, Anderson, et al., 2018; Wall et al., 2021). The response of organisms and the trajectories of ecosystems thus are becoming more contingent on previous disturbances (Brown et al., 2015; Guest et al., 2012; Hughes et al., 2019)—defined as “ecological memory” (Hughes et al., 2019). Ecological memory has principally been observed as a decrease in coral bleaching severity in a second marine heatwave compared to the level of bleaching observed in the initial heatwave, despite experiencing similar or greater heat exposure (Brown et al., 2015; Fisch et al., 2019; Gintert et al., 2018; Guest et al., 2012; Hughes et al., 2019; Pratchett et al., 2013). There may be several factors that lead to visual reductions in bleaching severity between events, including significant coral mortality resulting in a strong selection for robust coral host and Symbiodiniaceae genotypes (Burgess et al., 2021; Hughes, Kerry, et al., 2018; Quigley et al., 2022; Starko et al., 2023) and/or beneficial acclimatization through physiological plasticity (i.e., stress hardening) (Hackerott et al., 2021). While ecological memory may be an important strategy behind coral persistence (Brown et al., 2002, 2015; Hughes et al., 2019, 2021), our understanding is limited on the apparent acclimatization of reef‐building corals to consecutive heatwaves and its implications for the trajectory and resilience of coral reefs in the Anthropocene (Brown & Barott, 2022; Hackerott et al., 2021).

In corals that survive severe marine heatwaves, there is growing evidence of physiological acclimatization from the cellular (Wall et al., 2021) to ecosystem scale (Hughes et al., 2019). Beyond visual reductions in bleaching, antioxidant activity (Wall et al., 2021), host biomass (Thornhill et al., 2011; Wall et al., 2021), light‐harvesting pigments (Fisch et al., 2019), symbiont densities (Wall et al., 2021), and/or linear extension rates (Clarke et al., 2019) can be augmented relative to the first event. Alternatively, sensitization can occur—a compounding of stress precipitating from insufficient recovery periods (Brown & Barott, 2022)—materializing as bleaching during subsequent heatwaves (Dalton et al., 2020; Neal et al., 2017) or reduced growth (Baumann et al., 2019; Cantin & Lough, 2014) or fecundity for several years after the marine heatwave (Fisch et al., 2019; Levitan et al., 2014; Ward et al., 2002). As such, our understanding of the mechanisms allowing corals to recover from and develop resistance to consecutive marine heatwaves remains poorly understood, yet is key to predict the future of coral reefs in warming oceans.

**FIGURE 1:**
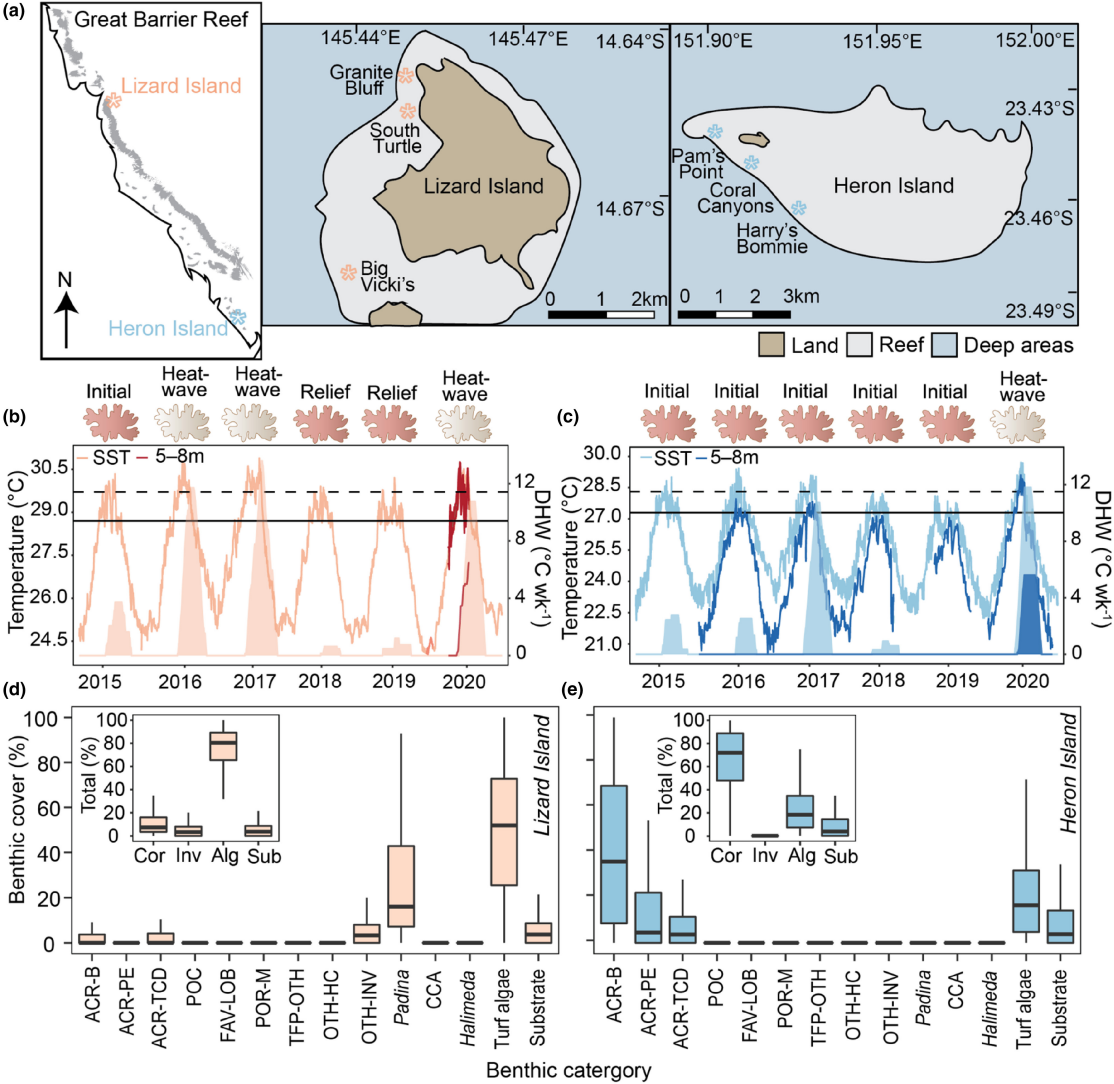
Study locations, thermal history, and benthic composition. (a) Map of the Great Barrier Reef, where insets detail the study sites at Lizard Island and Heron Island. Recent history (2015–2020) of temperature (left axis) and degree heating weeks (DHW; right axis) at (b) Lizard Island and (c) Heron Island. Corals were defined as an “initial” condition until a marine heatwave where significant coral bleaching was observed. Solid lines indicate daily (24‐h) mean temperatures for sea surface temperature (SST; light colors) from temperature recorded at a depth of 5–8 m (dark colors). Heat stress accumulation was estimated by degree heating weeks (shading) calculated from mean daily temperatures. Solid horizontal lines indicate the location's climatological maximum monthly mean (MMM) and dashed horizontal lines indicate the location's coral bleaching threshold (MMM + 1°C). Sea surface temperature data are from the National Oceanic and Atmospheric Administration (NOAA) Coral Reef Watch Virtual Stations. Measured benthic cover of (d) Lizard Island and (e) Heron Island in November and December 2019, respectively. Hard coral cover (%; minimum, 25th percentile, median, 75th percentile, and maximum) is displayed by functional group, with inset showing total benthic cover. ACR‐B, Acroporidae (branching); ACR‐PE, Acroporidae (plating/encrusting); ACR‐TCD, Acroporidae (tabular/corymbose/digitate); alg, algae; and sub, substrate (including bare rock, rubble and sand); CCA, crustose coralline algae; cor, hard coral; FAV‐LOB, Favidae‐Lobophyllidae; inv, other invertebrates; OTH‐HC, other hard coral; OTH‐INV, other invertebrates; POCI, Pocilloporidae; POR‐M, Poritidae (massive); TFP‐OTH, other thin, foliose or plating hard coral.

The goal of this study was to investigate whether ecological memory following consecutive marine heatwaves on the Great Barrier Reef (GBR) in 2016 and 2017 (Hughes et al., 2019) equates to identifiable physiological signatures of stress tolerance in a subsequent heatwave 3 years later (2020). Differential impacts of thermal stress and coral bleaching have naturally altered coral reef ecosystem configurations across the vast latitudinal expanse of the GBR (Hughes, Kerry, et al., 2018). Specifically, this study focused on two reefs: (1) Lizard Island, where severe coral bleaching and mortality (e.g., 30%–60% of all corals) were observed in 2016 and 2017, and (2) Heron Island, where minimal bleaching occurred in 2016 (<1% of corals) and 2017 (1%–10% of corals) (Figure 1) (Hughes et al., 2017). Within each location, two common genera of branching corals were investigated, *Pocillopora* and *Stylophora*, with coral colony size (juvenile: <10 cm, adult: >25 cm) used as a proxy for age to estimate if the corals were naïve or old enough to have experienced the 2016 and 2017 thermal stress events. Both reef systems experienced similar heat stress in a third marine heatwave in 2020 (Figure 1), which enabled us to test the hypotheses that previous exposure to heat stress lessens bleaching severity, and visual reductions in bleaching relate to increased survival. Further, we examined the genetic diversity, physiological performance, and trophic plasticity of naïve juveniles and hardened adults to determine whether selection for visually bleaching‐resistant genotypes stemming from a history of severe heat stress indicated beneficial acclimatization (Hughes et al., 2019) or resulted in trade‐offs with key physiological traits (i.e., growth, trophic strategies) (Cornwell et al., 2021), ultimately to improve our understanding of coral performance and resilience in the Anthropocene.

## 2 MATERIALS AND METHODS

### 2.1 Site selection, environmental records, and benthic community characterization

**FIGURE 2:**
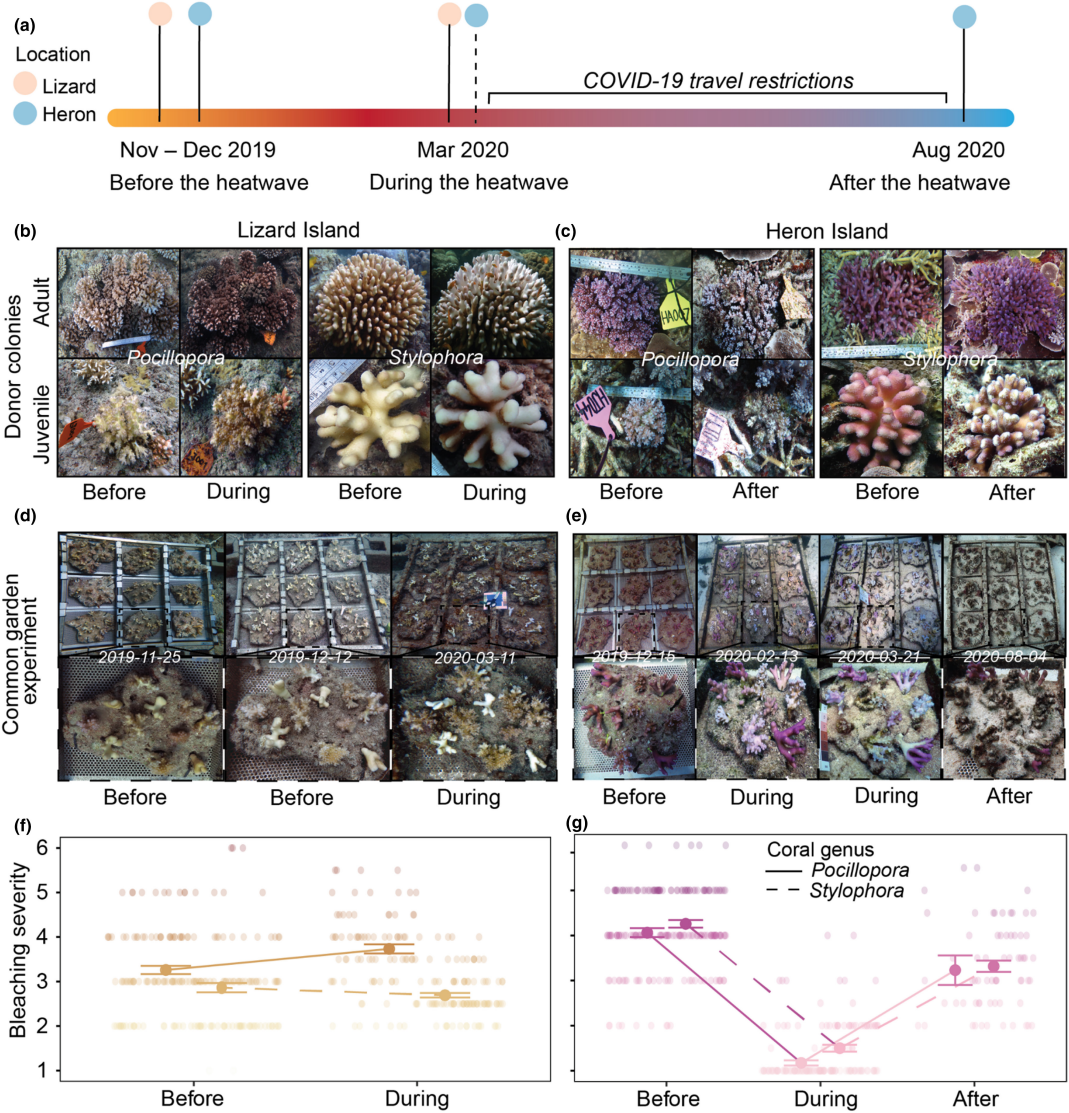
Representative images of field donor coral colonies and experimental fragments before, during, and after the 2020 marine heatwave across the Great Barrier Reef. (a) Timeline of the experiment indicates when each site was visited to conduct intensive reef‐wide surveys and physiological analyses (solid lines). Photographs were captured at Heron prior to travel restrictions (dashed line) to determine survival and bleaching severity during the heatwave. Donor colonies by the genus of adult (top row) and juvenile (bottom row) corals at (b) Lizard Island and (c) Heron Island over time. Common garden experiment detailing experimental trays (half pictured; top row) and the same tray of coral fragments (bottom row) at (d) Lizard Island and (e) Heron Island over time. Bleaching severity by coral genus at (f) Lizard Island and (g) Heron Island over time. Bleaching severity was visually determined using color standards (Siebeck et al., 2006), where (1) is most bleached and (6) is fully pigmented.

The study was performed from November 2019 to August 2020 on the GBR, Australia, at two distinct locations: Lizard Island (14.67° E, 145.44° S) and Heron Island (23.46° E, 151.95° S) (Figures 1 and 2, Figure S1). Three sites were investigated at each location: Big Vicki's, Granite Bluff, and South Turtle at Lizard Island and Coral Canyons, Harry's Bommie, and Pam's Point at Heron Island (Figure 1). The two locations differ significantly in their thermal stress history. During the thermal anomalies preceding this study (2016 and 2017), Lizard Island on the northern GBR experienced severe thermal stress, while Heron Island on the southern GBR experienced minimal stress (Figure 1) (Hughes et al., 2017). While sea surface temperature (SST) data from the National Oceanic and Atmospheric Administration (NOAA) Coral Reef Watch (CRW) Virtual Stations showed an accumulation of heat stress at both Lizard and Heron Island (Figure 1), in situ seawater temperatures (5–8 m) recorded from 2015 to 2019 at Heron Island indicated no cumulative in situ heat stress (Figure 1), aligning with observations of minimal coral bleaching in 2016 and 2017 (Hughes et al., 2017).

During the 2020 thermal anomaly, in situ seawater temperatures (HOBO Pendant UA‐001‐64, Onset) were recorded every 30 min at both Lizard Island (26 November 2019–13 March 2020) and Heron Island (26 July 2015–3 August 2020) at a depth of 5–8 m (*n* = 1–3 sensors per location) (Figure 1). Photosynthetically active radiation (Odyssey PAR sensor, Dataflow Systems Ltd) was integrated over 30‐min intervals across three sites in each location (depth of 5–8 m; *n* = 3 sensors per location) from November 26, 2019 to March 13, 2020 at Lizard Island and from November 26, 2019 to February 9, 2020 at Heron Island (Figure S2). PAR sensors were fitted with copper coating to prevent biofouling and were cross‐calibrated using a high‐precision photometer (DEFI‐L PAR logger; JFE Advantech Co. Ltd). Temperature loggers were cross‐calibrated with the in situ temperature data of the Australian Institute of Marine Science. The climatological maximum monthly mean (MMM), as determined from SST throughout the climatology period (1985–2001), is 28.7°C at Lizard Island and 27.3°C at Heron Island (NOAA CRW). Degree heating weeks were calculated using daily (24 h) mean temperatures following the equations in Brown et al. (2023).

Before the heatwave (November/December 2019), benthic community composition was determined along 8 × 30 m transects per site using the modified methodology of Bryant et al. (2017) (Table S1, for full details see Appendix S1). In order to assess isotope end members of planktonic food sources, plankton tows were conducted and seawater was collected and filtered (0.7 μm nominal pore size; Whatman GF/F) for particulate organic matter (POM) at each site (for full details see Appendix S1).

### 2.2 Common garden experiment and physiological analyses

Two widespread species, *Pocillopora damicornis* sensu lato and *Stylophora pistillata* sensu lato, were targeted for collection and used in the common garden experiment. These two species of branching coral were chosen based on the presence within both locations. Coral colony size was used as a proxy for age to determine whether the corals were old enough to have experienced the 2016 and 2017 thermal stress events. Based on published linear extension rates (Anderson et al., 2017; Burn et al., 2018), coral colonies that were >25 cm in diameter were defined as “adult,” whereas corals <10 cm in diameter were defined as “juvenile” (Figure 2). The size demographics of *Pocillopora* and *Stylophora* between the two study locations were determined before the 2020 heatwave (Figure S3; for full details see Appendix S1).

All coral fragments were collected using a hammer and chisel at a depth of 5–8 m using SCUBA. At Lizard Island, fragments were collected over a 10‐day period (15–25 November 2019) and at Heron Island, over an 8‐day period (4–12 December 2019) across the three sites (Figure 1). Colonies were selected randomly, with collections completed over an area of ~500 m^2^ at each site. Multiple fragments were collected from each coral colony (referred to throughout as a “donor” colony), with one fragment used for the common garden experiment and three fragments sacrificed for initial physiological, isotopic, and genetic analyses. A total of 72 donor *Pocillopora* colonies (12 per each colony size class per site) were tagged to serve as a living library for future studies (Figure 2). Due to time constraints, no *Stylophora* colonies were tagged. Fragments of a non‐symbiotic, scleractinian coral (*Tubastraea* cf. *coccinea*, *n* = 5 per location) were collected from low‐light underhangs (~10 m depth) as a local heterotrophic baseline.

Fragments used for the common garden experiment were standardized to a length of 5–8 cm (Figure 2). The base of each fragment was carefully leveled using a diamond wheel, and a small hole (~250 mm) was drilled using a Dremel attachment above the base of each fragment. A thin cable tie was threaded through the hole and fastened, creating a loop. Coral fragments were subsequently measured for net calcification via buoyant weight (Davies, 1989) and volumetric expansion through water displacement (Gutiérrez‐Heredia et al., 2015). Coral fragments were then attached to a live rock base to mimic the adjacent reef habitat using a cable tie that ran through the loop and a drilled depression in the rock (Figure 2). Between 9 and 12 fragments were randomly distributed between live rock bases (*n* = 18), intermixing genera and donor colony sizes (Figure 2). Each live rock was attached to a removable stainless‐steel tray, and fixed onto one of two metal structures (1.000 × 0.970 × 0.205 m) anchored to the benthos between 5‐ and 8‐m depth at Lizard Island (Granite Bluff) and at Heron Island (Harry's Bommie) (Figure 2). In total, 384 fragments were used in the common garden experiments (see Table 1 for sample sizes). The common garden experiment lasted 107 days at Lizard Island and 232 days at Heron Island. The longer duration at Heron Island was due to the COVID‐19 pandemic and associated travel restrictions canceling a scheduled expedition in March 2020.

**TABLE 1:** Survival and partial mortality across genera, location treatment, and holobiont, with the key elements of experimental design and replication.

Genus	Reef	Treatment	*n* by genus	Survival by genus (%)	Partial mortality by genus (%)	Holobiont	*n* by holobiont	Holobiont within genus (%)	Treatment	*n* by holobiont and treatment	Survival (%)	Collected from donor colony
*Pocillopora*	Lizard Island	Adult	48	81.3	23.7	*P. acuta* + *C. pacificum*	5	10.4	Juvenile	5	100.0	
						*P. bairdi* + *C. pacificum*	24	50.0	Adult	13	84.6	
									Juvenile	11	81.8	
		Juvenile	48	79.2	25.1	Pnew (rel *P. verrucosa*) + *C. pacificum*	3	6.3	Adult	2	0.0	
									Juvenile	1	0.0	
						*P. verrucosa* (OPI40) + *C. pacificum*	16	33.3	Adult	9	88.9	
									Juvenile	7	85.7	
*Stylophora*		Adult	48	100.0	5.8	*S. pistillata* + C8 group	48	100.0	Adult	24	100.0	
		Juvenile	48	100.0	5.4				Juvenile	24	100.0	
*Pocillopora*	Heron Island	Adult	48	20.8	90.4	*P. damicornis* + *C. latusorum*	7	14.6	Adult	3	66.7	2
									Juvenile	4	50.0	1
						*P. damicornis* + C33a	38	79.2	Adult	19	5.3	11
		Juvenile	48	14.6	90.9				Juvenile	19	0.0	3
						Pnew (rel *P. damicornis*) + C33a	3	6.3	Adult	2	0.0	
									Juvenile	1	0.0	
*Stylophora*		Adult	48	50.0	60.7	*S. pistillata* + C78a	25	52.1	Adult	9	77.8	
									Juvenile	16	81.3	
						*S. pistillata* + C8 group	12	25.0	Adult	10	50.0	
		Juvenile	48	58.3	65.2				Juvenile	2	50.0	
						*S. pistillata* + C35a group	9	18.8	Adult	3	0.0	
									Juvenile	6	0.0	

### 2.3 Physiological measurements

#### 2.3.1 Bleaching severity and survival

Survival was evaluated as the binary state, with fragments devoid of living tissue and covered with turf algae defined as dead. On all surviving fragments, partial mortality was estimated visually, with any areas covered with turf algae estimated as a percentage of the entire fragment. In March 2020 at Heron Island, survivorship was quantified from photographs taken in situ and partial mortality was not estimated (Figure 2, Figure S1). Coral color, a proxy for coral bleaching severity, was assessed up to three times: (1) before the heatwave at Lizard Island (25 November 2019) and at Heron Island (15 December 2019), (2) during the peak of the heatwave at Lizard Island (11 March 2020) and at Heron Island (21 March 2020), and (3) after the heatwave at Heron Island only (4 August 2020) using coral color cards (Siebeck et al., 2006) (Figure 2). Before (both locations) and during the heatwave (Heron Island), coral bleaching was estimated across the entire fragment from photographs taken in situ. During (Lizard Island) and after (Heron Island) the heatwave, the front and the back of the coral were each given a score ex situ and averaged due to evident patterns in bleaching.

#### 2.3.2 Growth

To account for any variability in starting measurements, the relative change in buoyant weight and volumetric expansion was determined by:

$$\begin{array}{c} \text{Relative change}\;\left(\%\Delta \;{\mathrm{day}}^{-1}\right)=\left(\left(M_{\text{final}}–M_{\text{initial}}\right)÷M_{\text{initial}}\times 100\right)\\ \;÷\mathrm{no}.\;\text{days}, \end{array}$$

where *M*_final_ is the measurement taken in March 2020 (Lizard)/August 2020 (Heron), *M*_initial_ is the measurement taken in November 2019 (Lizard)/December 2019 (Heron) and number of days (no. days) is the length of the experiment.

#### 2.3.3 Photochemical yield

Dark‐adapted maximum quantum yield (*F*_v_/*F*_m_) was assessed on all surviving fragments using a Diving‐PAM (Walz GmbH) approximately 1 h after sunset during the heatwave at Lizard Island (March 2020) and after the heatwave at Heron Island (August 2020). Measurements were made using the Diving‐PAM 5‐mm diameter fiber‐optic probe at a standardized distance of 5 mm above the coral tissue after *F*_o_ stabilized. Three random sections that appeared uniform in color were measured on each fragment, and if corals appeared partially bleached, three additional measurements were taken within the bleached area (*n* = 3–6 per fragment), with photochemical yield averaged across the entire fragment. Following all living measurements, corals were rinsed with 0.7 μm filtered seawater and frozen at −20°C, with a subset of fragments (*n* = 6–20) analyzed for physiological and isotopic analyses.

#### 2.3.4 Genetics, host tissue, and symbiont concentrations

A small chip (2–3 mm) of each fragment was preserved in 100% ethanol and stored at −80°C prior to genetic analyses of both the coral host and its endosymbionts (for full details see Appendix S1). Briefly, our objective was to match collected specimens to known host and symbiont species descriptions. Coral species identification for *Pocillopora* was confirmed with a genetic assay using the mitochondrial ORF region (Flot & Tillier, 2007; Schmidt‐Roach et al., 2013). No such assay was available for *Stylophora*, and all specimens matched the description of *S. pistillata* (Veron, 1993). For the symbiont, baseline ITS2‐type identification was done for both *Pocillopora* and *Stylophora* specimens. The chloroplast psbA minicircle noncoding region was sequenced (cf. LaJeunesse & Thornhill, 2011) and blasted against the recently described symbiont species *Cladocopium pacificum* and *Cladocopium latusorum* (Turnham et al., 2021) from *Pocillopora* coral species.

Before the heatwave, host soluble protein concentrations, endosymbiont cell densities, and chlorophyll *a* content were determined from one coral fragment, and host and symbiont δ^13^C, δ^15^N, %C, C:N ratio, δ^13^C_host‐symbiont_, and δ^15^N_host‐symbiont_ (often referred to as Δ^13^C and Δ^15^N) were determined from another fragment from each donor colony. During/after the heatwave, protein densities, endosymbiont cell densities, chlorophyll *a* content, host δ^13^C, δ^15^N, %C and C:N were determined either: (i) from the original fragment used in the common garden experiment, or (ii) for *Pocillopora* at Heron Island, a new fragment from the original donor colony on the reef due to significant mortality of experimental fragments (Table 1). Coral tissue was removed from skeletons using a waterpik with 50 mL of 0.1 M phosphate‐buffered saline solution. The tissue slurry was centrifuged at 4°C once for 5 min at 2500 *g* to separate host tissue and the intracellular endosymbiont cells. For isotopic analyses, the separated host tissue slurry and symbiont pellet (resuspended in filtered seawater) were repeatedly centrifuged until no symbiont material was visible in the host slurry. The cleaned host and symbiont fractions were briefly acidified with drops of HCl (1 M) to remove any skeletal fragments, frozen at −80°C, and freeze‐dried (ScanVac CoolSafe) prior to isotope analyses. Protein densities were determined spectrophotometrically using the empirical equations of Whitaker and Granum (1980). Endosymbiont cell densities were determined by microscopy using a hemocytometer and counting three replicate aliquots of the cell suspension per fragment. Endosymbiont photopigments were extracted in 100% acetone for 24 h and concentration of chlorophyll *a* was determined via absorbance at 630, 663, and 750 nm using the equation (Jeffrey & Humphrey, 1975):

$$\text{Chlorophyll}\;a=\left(11.43\times \left(A663-A750\right)\right)-\left(0.64\times \left(A630-A750\right)\right)$$

Protein and symbiont densities were standardized to surface area (cm^2^), which was determined using the wax‐dipping technique (Holmes, 2008), whereas pigment concentrations were standardized to both surface area and symbiont densities.

#### 2.3.5 Host and symbiont stable isotopes

Analysis of carbon and nitrogen elements and stable isotope values (δ^13^C and δ^15^N) of coral host tissues, symbionts, non‐symbiotic coral *T*. cf. *coccinea*, and plankton was carried out with an Elementar PrecisION isotope ratio mass spectrometer (IRMS) coupled to an Elementar Vario Isotope Cube at the Stable Isotope Geochemistry Laboratory at the University of Queensland. Host tissues (adults and juveniles) were assessed three times: (1) before (*n* = 48; Lizard and Heron), (2) during (*n* = 42; Lizard only), and (3) after the heatwave (*n* = 30; Heron only). Zooplankton (three size fractions) were assessed twice including before (*n* = 35 Lizard, *n* = 30 Heron) and during (*n* = 18; Lizard only); whereas symbiont (*n* = 43; Lizard and Heron) and *T*. cf. *coccinea* (*n* = 5; Lizard and Heron) were carried out only before the heatwave (Table S3). Repeated measurements of laboratory standards resulted in analytical precision of 0.05‰ for δ^13^C and <0.15‰ for δ^15^N (USGS40, USGS41a, USGS43) with a subset of host and symbiont samples (*n* = 28) run in duplicate or triplicate (Table S2). Repeated samples had a standard error of 0.09‰ and 0.03‰ and standard deviation of 0.13‰ and 0.05‰ for δ^15^N and δ^13^C, respectively. Glass fiber filters (POM; 0.7 μm nominal pore size) were analyzed for δ^13^C and δ^15^N (before heatwave; *n* = 27 Lizard, *n* = 32 Heron) with a Sercon 20–22 IRMS and GSL inlet at the Central Analytical Research Facility at Queensland University of Technology. For the filters, the primary standards used were IAEA‐N1 and IAEA CH‐6 plus two soil and one plant internal lab standards. Stable isotope data are reported in the delta (*δ*) notation per mil (‰) relative to standard materials (Vienna Pee‐Dee Belemnite [V‐PDB] and atmospheric N_2_ [Air] for C and N, respectively):

$$\delta^{13}C\;\text{or}\;\delta^{15}N=\left[\left(R_{\text{sample}}/R_{\text{standard}}\right)-1\right]\times 1000,$$

where *R* is the ratio of the heavy‐to‐light isotope (i.e., ^13^C/^12^C or ^15^N/^14^N).

#### 2.3.6 Statistical analyses

All statistical analyses were done using R version 4.0.0 software (R Core Team, 2021), and graphical representations were produced using the package *ggplot2* (Wickham, 2016). Differences of in situ PAR were explored between the locations (Lizard, Heron) using linear models for the 3‐month period from November 2019 to February 2020. Differences in benthic community composition were explored by location using permutational multivariate analysis of variance (PERMANOVA) using the adonis function in the *vegan* package (Oksanen et al., 2013). Resemblance matrices were obtained using the Bray–Curtis dissimilarity and 9999 permutations. The interactive effects of location, coral genera (*Pocillopora* and *Stylophora*), and coral colony size (cm) (six discrete groups: <10, 11–20, 21–30, 31–40, 41–50, >51) were explored before the heatwave using a generalized linear mixed effects (glmer) model with Poisson distribution. The interactive effects of location, coral genera, and colony size (adult: >25 cm, juvenile: <10 cm) were explored for the relative change in net calcification (%Δ BW day^−1^) and volumetric expansion (%Δ g day^−1^) using linear mixed effects (lme) models with site as a random effect (Bates et al., 2015). Similarly, for physiological metrics, the interactive effects of categorical predictors location, coral genera, colony size, and time (before, during/after) were explored for survival, bleaching severity, photosynthetic efficiency, endosymbiont density, chlorophyll *a* concentrations, host soluble protein, coral host δ^15^N, δ^13^C, and %C using lme models with site as a random effect. Using data only collected before the heatwave, the interactive effects of: (i) location, coral genera, and colony size were explored on δ^13^C_host‐symbiont_ and δ^15^N_host‐symbiont_ (Δ^13^C, Δ^15^N) and symbiont δ^15^N, δ^13^C, and %C, (ii) location and size fraction (65–153 μm, 153–300 μm, and >300 μm) were assessed for plankton δ^13^C and δ^15^N, and (iii) the individual effect of location on POM δ^13^C and δ^15^N using lme models with site as a random effect. Stable isotope data are presented as mean ± SD and as standard ellipse areas (isotopic niche) comprising 40% of each group data set (Jackson et al., 2011). A linear model was used to test the relationship between host protein and δ^13^C_host‐symbiont_ (Δ^13^C), two common metrics for coral heterotrophy, to investigate trophic baselines before bleaching. The significance of fixed effects and their interactions was determined using an analysis of variance with a type III error structure using the Anova function in the *car* package (Fox et al., 2012). Significant interactive effects were followed by pairwise comparison of estimate marginal means using the *emmeans* package with Tukey HSD adjusted *p* values (Lenth et al., 2018). All data met assumptions (homogeneity of variance, normality of distribution) through graphical analyses of residual plots.

Differences in coral multivariate phenotypes were analyzed separately for each time point (before, during, and after) using PERMANOVA and principal component analysis (PCA), with the fixed effects of location, coral genera, and colony size using the adonis and rda functions in the *vegan* package, respectively (Oksanen et al., 2013). Resemblance matrices were obtained using the Bray–Curtis dissimilarity and 9999 permutations.

## 3 RESULTS

### 3.1 Regional differences in environmental conditions, benthic community composition, and coral size structure

During the 2020 thermal anomaly, in situ heat stress was higher at Lizard Island (6.3°C week^−1^) than at Heron Island (5.6°C week^−1^) (Figure 1). Heat stress began accumulating in mid‐January and peaked in mid‐March, at which time cyclonic activity resulted in the rapid dissipation of heat stress across both locations (2.5°C decline in <2 weeks; Figure 1). PAR (μmol quanta m^−2^ s^−1^; 24‐h mean ± SE) was slightly higher at Lizard (147 ± 4) than at Heron (133 ± 4) across the time period where in situ data were concurrently recorded between the locations (November 2019–February 2020) (*F* = 4.96, *p* = .027) (Figure S2).

Prior to the heatwave, benthic community composition was significantly different between locations (pseudo *F* = 646.13, *p* < .0001). Notably, total hard coral cover was significantly lower at Lizard Island (10.6% ± 0.41%) than at Heron Island (66.0% ± 0.98%) (Figure 1). Acroporids were the most abundant reef‐building corals at both locations, with tabular/corymbose/digitate acroporids (ARC‐TCD) (3.6% ± 0.22%) the most abundant at Lizard Island and branching acroporids (ACR‐BRA) (41% ± 1.2%) the most abundant at Heron Island (Figure 1). At Lizard Island, total algae cover was the most prominent benthic category (75.7% ± 0.68%), with turf algae (50.2% ± 0.98%) and fleshy macroalgae *Padina* (25.5% ± 0.86%) the most abundant. At Heron Island, total algae cover was almost three times less than that at Lizard Island (22.8% ± 0.72%) and was almost entirely composed of turf algae (21.7% ± 0.71%). Other invertebrates, including soft corals and sponges, were more abundant at Lizard Island than at Heron Island (6.4% ± 0.4% vs. 0.9% ± 0.12%, respectively).

Coral colony size distribution was significantly influenced by the three‐way interaction between location, genus, and colony size class (*χ* ^2^ = 81.5, *p* < .0001). Pairwise analyses revealed Lizard Island had significantly more *Pocillopora* colonies across the median size classes (11–20, 21–30, 31–40 cm) than Heron Island (*p* < .005), and no difference in the most abundant smallest size class (<10 cm) (*p* = .13) or least abundant largest classes (41–50, >51 cm) (*p* > .96) (Figure S3). For *Stylophora*, there were no significant differences between colony size classes between locations (*p* > .21), with the exception of 21–30 cm, where Heron had significantly more colonies within that size class than Lizard (*p* < .009) (Figure S3).

### 3.2 Coral species and Symbiodiniaceae associations across the Great Barrier Reef

Genetic identification indicated that the collected *Pocillopora* specimens comprised several distinct ORF genotypes that were consistent with several previously identified *Pocillopora* species (GenBank accession numbers OR837528 ‐ OR837623; Figure S4, cf. Schmidt‐Roach et al., 2014). At Lizard Island, the total 48 specimens examined (Table 1) consisted of: 5 *Pocillopora acuta* (all <10 cm), 24 *Pocillopora bairdi*, 16 *Pocillopora verrucosa*, and a further 3 were close relatives of *P. verrucosa* (2 bp difference, low bootstrap support; Figure S4). The latter three were conservatively grouped with *P. verrucosa* for physiological analyses. At Heron Island, 45 specimens were identified as *P. damicornis* with a further three samples derived from the main *P. damicornis* clade (1 bp difference; Figure S4) and were conservatively grouped with *P. damicornis* in subsequent physiological analyses.

Based on ITS2, *Pocillopora* specimens at Lizard Island hosted either *Cladocopium* ITS2‐type “C1‐c” (*n* = 2), “C1‐cc” (*n* = 2), “C1‐c‐d” (*n* = 10), or “C1‐c‐d‐t” (*n* = 34), all of which confidently assigned to *C. pacificum* based on their psbA sequence (Turnham et al., 2021) (Table S4). One specimen contained *Durusdinium trenchii* as a background symbiont and one specimen appeared to contain a mix of *C. pacificum* and *C. latusorum* based on psbA sequences. At Heron Island, *P. damicornis* specimens hosted either *Cladocopium* ITS2‐type “C1b‐c, 42a” (*n* = 5) or “C33a” (*n* = 33; cf. Sampayo et al., 2009). Samples with ITS2‐type “C1b‐c, 42a” corresponded to *C. latusorum* based on the psbA sequences, while those with “C33a” represented a separate group that could not be aligned to either *C. latusorum* or *C. pacificum*. The divergence of the “C33a” psbA minicircle noncoding regions corresponds with previous assertions that this particular ITS2‐type represents a distinct species (Sampayo et al., 2009). There were no specific site‐ or colony size‐related patterns found in the distribution of these two symbionts at Heron. Importantly, there was no overlap in either host *Pocillopora* or associated symbiont *Cladocopium* species between Heron and Lizard Island.

All specimens matched the description of *S. pistillata*, but noting that suggestions exist in the literature that this coral may comprise multiple species (Kaniewska & Sampayo, 2022; Keshavmurthy et al., 2013). All 48 *Stylophora* specimens from Lizard Island contained either *Cladocopium* ITS2‐ type “C8” (*n* = 11) and “C8‐b” (*n* = 25) (cf. Tonk et al., 2014) or “C8‐unk” (*n* = 12; containing a co‐dominant sequence “unk” in addition to C8 that could not be identified/sequenced here). At Heron Island, *Stylophora* specimens contained either *Cladocopium* ITS2‐type “C8a” (*n* = 12) and “C78a” (*n* = 25) or “C35a” (*n* = 9) (Table S4). Some site differences were observed, with “C8a” at higher abundance at Pam's Point and “C78a” more abundant in other sites (Coral Canyons and Harry's Bommie). Due to the persistence of C8 in the four types “C8, ‘C8‐a’, C8‐b” and “C8‐unk” and their regional occurrence along the GBR (Sampayo et al., 2007; Tonk et al., 2014), these four types were here considered putative population variants of a likely species (referred to herein as *Cladocopium* “C8 group”) for subsequent physiological analyses. *Stylophora* containing “C35a” were not included in analyses because these corals did not survive the heatwave and the effects of time (before, during/after) could not be explored. Only complete chloroplast psbA sequences were deposited to GenBank (accession numbers OR887551 ‐ OR887595), and partial sequences can be accessed as a fasta file from https://github.com/imkristenbrown/Coral‐ecological‐memory‐from‐heatwaves.

### 3.3 Greater survival and less bleaching in corals with ecological memory

**FIGURE 3:**
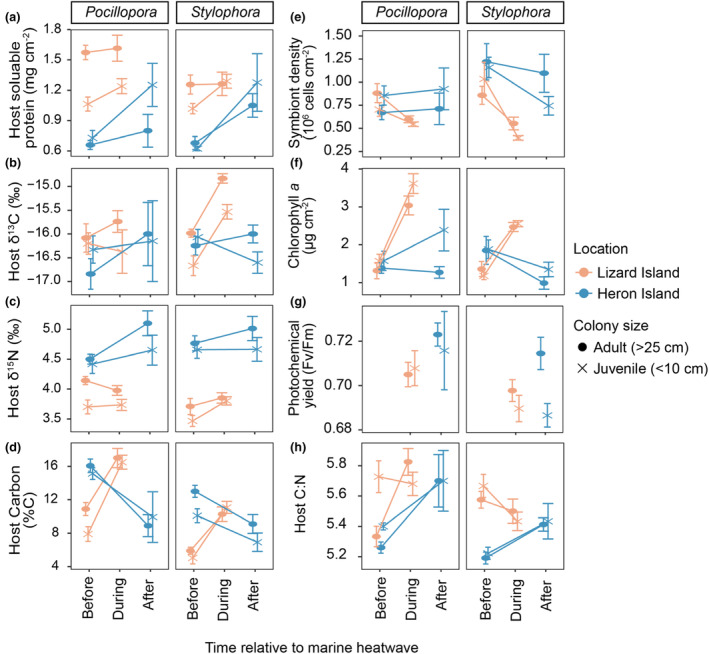
Physiological responses of *Pocillopora* and *Stylophora* before, during, and after the marine heatwave in 2020. (a) Host soluble protein concentrations, (b) host carbon stable isotopes (δ^13^C), (c) host nitrogen stable isotopes (δ^15^N), (d) host carbon, (e) symbiont cell density, (f) chlorophyll *a* content, (g) photochemical yield (*F*_v_/*F*_m_), and (h) host C:N. Points represent mean ± SE by region and colony size.

Coral color, or bleaching severity, was influenced by the interaction of location, coral genus, and time (*F* = 9.38, *p* = .002). Prior to the heatwave (November/December 2019), corals of both genera at Heron were more pigmented than conspecifics at Lizard (*p* < .0001) (Figure 2). At the peak of the heatwave (March 2020), Heron corals were approximately two times less pigmented than corals at Lizard (*p* < .0001), with contrasting patterns between genera within locations (Figure 2). At Lizard Island, *Stylophora* were less pigmented than *Pocillopora*, whereas at Heron Island, the opposite was observed, with *Pocillopora* more bleached than *Stylophora* (*p* < .0001) (Figure 2). Interestingly, photochemical yield (*F*_v_/*F*_m_) remained high in both *Pocillopora* (0.71 ± 0.02) and *Stylophora* (0.69 ± 0.02) at Lizard Island at the peak of the heatwave, and did not differ from the photochemical yield of surviving corals 5 months after peak heat stress at Heron Island (*Pocillopora*: 0.70 ± 0.08; *Stylophora*: 0.70 ± 0.03) (Figure 3). After the heatwave (August 2020), Heron Island corals regained some pigmentation, but were still significantly less pigmented compared to those at pre‐heatwave levels (*p* < .0001) (Figure 2).

Assessments of survival at the peak of the heatwave (March 2020) revealed a greater proportion of corals were alive at Lizard Island (90.1%) than at Heron Island (82.6%) (*χ* ^2^ = 32.6, *p* < .0001). In surviving corals at Lizard, *Pocillopora* experienced greater partial mortality than *Stylophora* (*χ* ^2^ = 20.9, *p* < .0001), where mean fragment mortality was 24.4% in *Pocillopora* and 5.4% in *Stylophora* (Table 1). Five months after the heatwave, 33.9% of fragments remained alive at Heron Island, consisting of over half of all *Stylophora* (54.2%), but only 13.5% of *Pocillopora* (*χ* ^2^ = 17.6, *p* < .0001) (Table 1). Corals that survived at Heron experienced extreme partial mortality, with mean fragment partial mortality of 90.6% in *Pocillopora* and 62.9% in *Stylophora* (Table 1). At the holobiont level, generally, more rare species did not survive the heatwave (Table 1).

Trends observed for experimental fragments matched the bleaching patterns for both tagged *Pocillopora* colonies (Figures S5 and S6) and reef‐wide bleaching patterns in both *Pocillopora* and *Stylophora* during the heatwave at Lizard (Figure S7). Interestingly, a positive relationship was found between bleaching prevalence and coral colony size in *Pocillopora* colonies (*R* ^2^ = .309); however, no correlation between bleaching prevalence and coral colony size was found for *Stylophora* (*R* ^2^ = .0) (Figure S7). At Heron, patterns in survival and partial mortality within tagged *Pocillopora* colonies generally followed what was observed for experimental fragments after the marine heatwave (Figure S8).

### 3.4 Greatest calcification rates in *Pocillopora* from Lizard Island despite previous exposure to heatwaves

**FIGURE 4:**
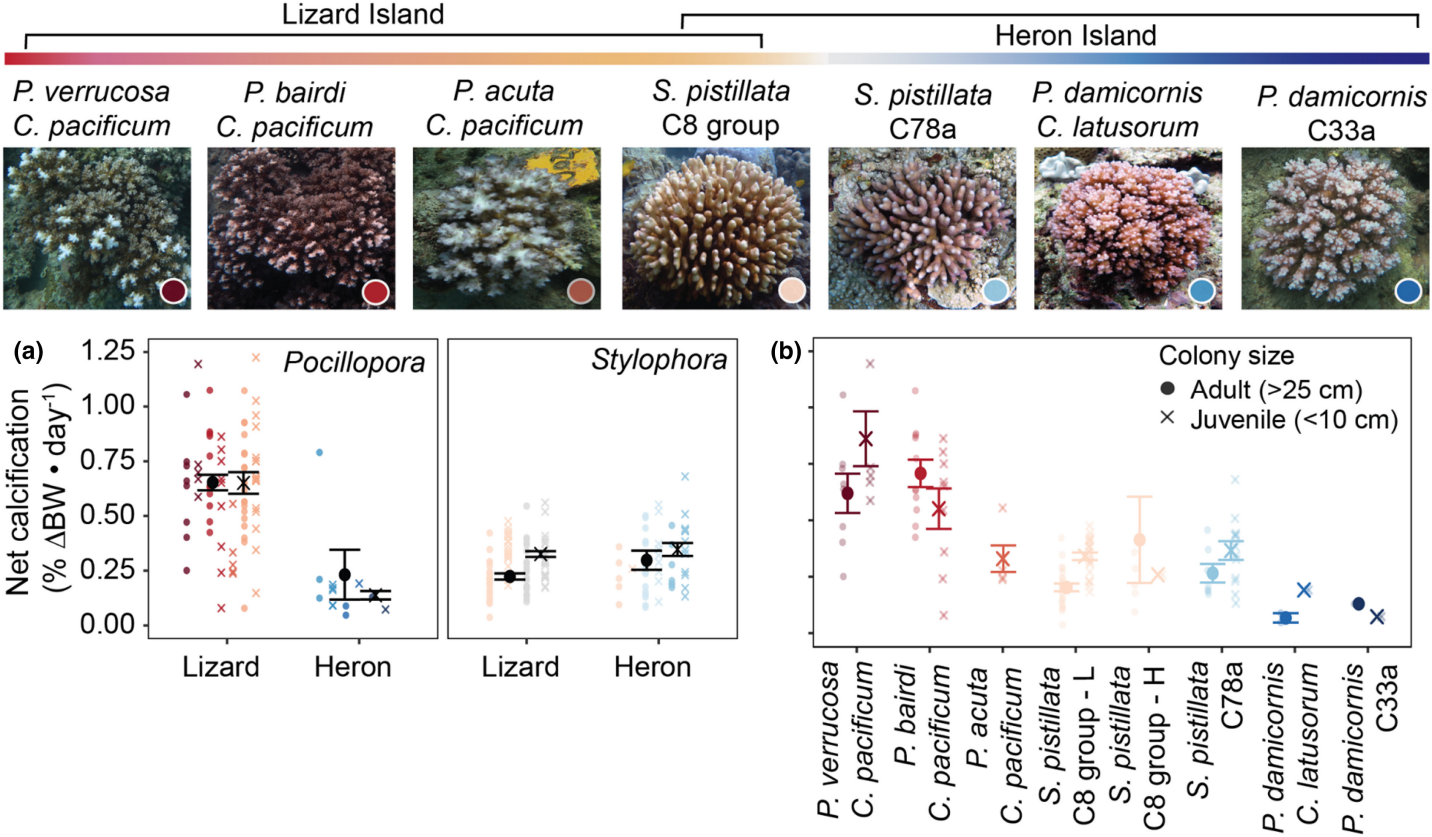
Coral calcification across the Great Barrier Reef by location, colony size, and holobiont. Representative images of coral holobionts from Lizard Island and Heron Island. (a) Relative change in buoyant weight (mean ± SE) by location, coral genus, and colony size. (b) Relative change in buoyant weight (mean ± SE) by colony size and holobiont (bottom). Points represent individual coral fragments.

Relative change in net calcification (%Δ BW day^−1^) was influenced by the interaction between location and coral genus (*χ* ^2^ = 60.3, *p* < .0001) as well as coral genus and colony size (*χ* ^2^ = 4.78, *p* = .028). Pairwise comparisons revealed that adult corals of the genus *Stylophora* calcified significantly less than juveniles (*p* = .02) regardless of location, whereas no differences were found by colony size for *Pocillopora* (*p* = .38) (Figure 4). Further, regardless of coral colony size, *Pocillopora* at Lizard calcified significantly more than conspecifics at Heron (*p* = .0056), whereas no differences were found between locations for *Stylophora* (*p* = .64) (Figure 4). Within locations, *Stylophora* calcified significantly less than *Pocillopora* at Lizard (*p* < .0001), whereas no difference was found between genera at Heron (*p* = .08) (Figure 4). Similarly, for volumetric expansion (%Δ day^−1^), the interaction between coral genus and location emerged as significant (*χ* ^2^ = 58.7, *p* < .0001), with *Pocillopora* at Lizard expanding significantly more than *Stylophora* within the same location (*p* < .0001) as well as conspecifics at Heron (*p* = .008) (Figure S9).

While insufficient sample sizes prevented statistical analyses across specific holobiont combinations (Table 1), significant variability in calcification rates were observed within the *Pocillopora* species complex at Lizard Island (Figure 4). Smaller colonies exhibited a trend toward higher calcification and expansion rates than larger colonies, particularly for *P. verrucosa* (Figure 4, Figure S9). Generally, closely related species showed similar rates, with *P. verrucosa* and *P. bairdi* exhibiting higher calcification rates than *P. actua* within Lizard Island (Figure 4), the latter being more related to *P. damicornis* at Heron Island (Figure S4).

### 3.5 Energy reserves elevate in response to heatwaves

Host soluble protein concentration was significantly influenced by the three‐way interaction between location, coral genus, and colony size (*χ* ^2^ = 5.1, *p* = .024). Before the 2020 heatwave, adult corals of the genus *Pocillopora* at Lizard Island (1.59 ± 0.07 mg cm^−2^) had significantly higher protein densities than juveniles (0.99 ± 0.09 mg cm^−2^) (*p* < .0001), with no differences between colony sizes observed for *Stylophora* at Lizard (*p* = .5) or for either genus at Heron (*p* > .05) (Figure 3a). At Lizard Island, adult corals of both *Pocillopora* (1.59 ± 0.07 mg cm^−2^) and *Stylophora* (1.26 ± 0.07 mg cm^−2^) had significantly greater protein densities than conspecifics at Heron (*Pocillopora*: 0.729 ± 0.09, *Stylophora*: 0.87 ± 0.09) before the 2020 heatwave (Figure 3a). Interestingly, a significant interaction emerged between colony size and time (*χ* ^2^ = 5.6, *p* = .018), where regardless of species, juveniles had higher protein densities during/after thermal stress to levels indistinguishable from adults (*p* = .76).

Coral host δ^13^C (mean ± SD) showed a marginally insignificant interaction among location, coral genus, and time (*χ* ^2^ = 3.5, *p* = .059) (Table S3). Interestingly, during the heatwave, *Stylophora* from Lizard Island displayed elevated host δ^13^C values (−15.2‰ ± 0.6‰) in contrast to before (−16.3‰ ± 0.6‰) (*p* < .0001), with adult *Stylophora* showing the greatest change (−14.8‰ ± 0.3‰) (Figure 3b). The individual effect of colony size emerged as marginally significant (*χ* ^2^ = 3.95, *p* = .047), with slightly lower host δ^13^C values in juveniles (−16.3‰ ± 0.9‰) than in adults (−16.0‰ ± 0.9‰) (Figure 3b); this was driven by lower host δ^13^C in juvenile *Stylophora* (−16.7‰ ± 0.7‰) than in adults (−16.0‰ ± 0.3‰) at Lizard Island before the heatwave. Interestingly, corals that ultimately did not survive the heatwave had lower symbiont δ^13^C values before the heatwave (−17.0‰ ± 1.4‰) than symbiont δ^13^C in surviving corals (−16.0‰ ± 0.8‰) (Figure S10).

Host δ^15^N was influenced by the interaction between location and coral genus (*χ* ^2^ = 9.1, *p* = .003) and location and time (*χ* ^2^ = 5.4, *p* = .020) (Figure S11, Table S3). Both *Pocillopora* (4.6‰ ± 0.4‰) and *Stylophora* (4.7‰ ± 0.5‰) at Heron Island had higher host δ^15^N values (mean ± SD) than those at Lizard Island (3.9‰ ± 0.3‰, *p* = .006 and 3.7‰ ± 0.4‰, *p* = .001, respectively) (Figure 3c). Similarly, host δ^15^N values of a non‐symbiotic, scleractinian coral (*Tubastraea* cf. *coccinea*) were higher at Heron (range 6.3‰–8.5‰) than at Lizard Island (range 5.0‰–6.3‰) (Figure S14, Table S3). Location‐specific host δ^15^N differences were maintained over time, with no changes at Heron (*p* = .138) or Lizard Island (*p* = .706) during/after heat stress (Figure 3c).

Host carbon content (%C) was affected by the three‐way interaction among location, coral genus, and time (*χ* ^2^ = 5.145, *p* = .023) (Figure 3d). Host %C increased during the heatwave at Lizard Island in both *Pocillopora* (from before to during the heatwave; 9.5%–16.7%) and *Stylophora* (5.5%–10.7%), while at Heron, in both *Pocillopora* (15.7%–9.4%) and *Stylophora* (11.6%–8.1%) it decreased after the heatwave (Figure 3d). Before the heatwave, symbiont %C (mean ± SD) was nearly two times higher at Heron Island (41.5% ± 12.6%) than at Lizard Island (22.8% ± 8.9%) (*χ* ^2^ = 6.1, *p* = .01363) (Figure S12).

Symbiont density showed a significant interaction between location and time (*χ* ^2^ = 4.9, *p* = .03) as well as coral genus and time (*χ* ^2^ = 5.5, *p* = .02). At Lizard, symbiont densities were significantly reduced during the heatwave regardless of species (*p* < .0001), whereas at Heron, there were no differences in symbiont densities before the heatwave or after (*p* = .32) (Figure 3e). Further, symbiont density was significantly influenced by the three‐way interaction among location, coral genus, and colony size (*χ* ^2^ = 4.8, *p* = .028), with pairwise comparisons revealing adult corals of the genus *Stylophora* had significantly higher symbiont densities at Heron than conspecifics at Lizard, regardless of time (*p* = .04) (Figure 3e). Chlorophyll *a* (pg cm^−2^) concentrations showed several significant two‐way interactions including between location and time (*χ* ^2^ = 65.2, *p* < .0001), where chlorophyll *a* concentrations were significantly increased during the heatwave at Lizard Island (*p* < .0001) (Figure 3f). Conversely at Heron Island, there were no differences in chlorophyll *a* concentrations before or after the heatwave (*p* = .35) (Figure 3f). Further, a significant interaction between coral genus and colony size (*χ* ^2^ = 4.8, *p* = .03) revealed significantly greater chlorophyll *a* concentrations in juveniles of the genus *Pocillopora* than in adults, regardless of time or location (*p* = .0009) (Figure 3f).

### 3.6 Variability in environmental history and local resources influence trophic state

**FIGURE 5:**
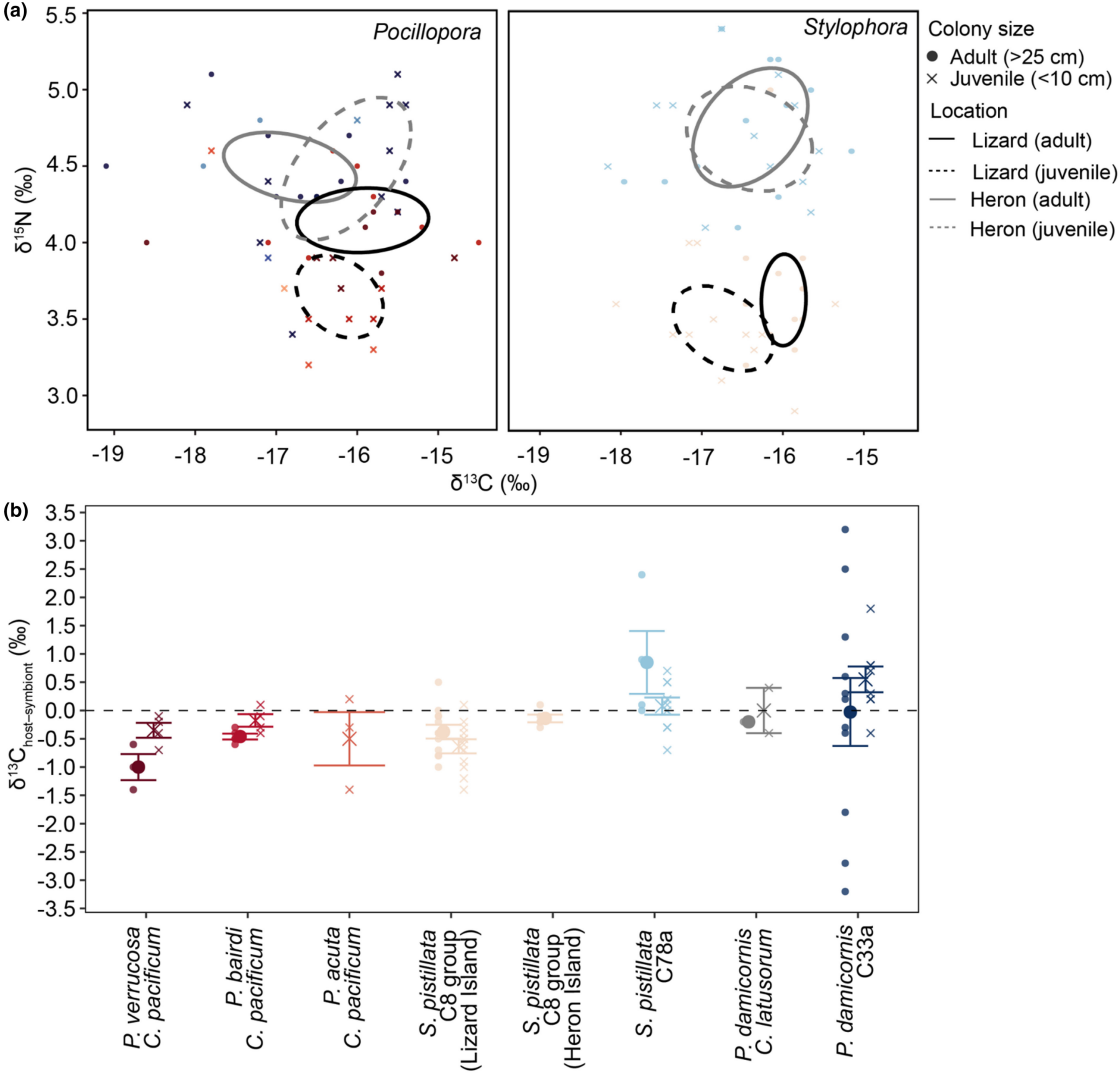
Coral tissue stable isotopes from before the 2020 marine heatwave on the Great Barrier Reef. (a) Isotopic niches of coral hosts *Pocillopora* and *Stylophora* of adult and juvenile size classes from Heron and Lizard Island. Isotopic niches represent the standard ellipse areas comprising core data (40%). Differences in (b) carbon stable isotopes of coral host versus symbiont (δ^13^C_host‐symbiont_, also Δ^13^C) in adults and juveniles before the marine heatwave (Lizard—four left genotypes, Heron—four right genotypes; dashed horizontal line depicts zero on the *y*‐axis).

Before the heatwave, adult and juvenile host isotopic niches occupied distinct isotopic space for both coral genera at Lizard Island but not at Heron Island (Figure 5a), whereas during/after the heatwave, host isotopic niches generally became more separated across genera and locations (Figure S13). The POM isotopic niche was distinct from each plankton isotopic niche at Heron Island, but at Lizard Island, the POM isotopic niche overlapped with the two larger size fractions of plankton plus the non‐symbiotic coral niche (Figure S14). The non‐symbiotic, scleractinian coral used as a local heterotrophic baseline (*Tubastraea* cf. *coccinea*) had similar mean δ^15^N (5.6‰) as the largest plankton size fraction (5.7‰, >300 μm) and POM (5.3‰) at Lizard Island (Figures S11 and S14, Table S3). At Heron Island, the non‐symbiotic heterotrophic coral had higher mean δ^15^N (7.3‰) than the largest plankton size fraction (5.5‰, >300 μm) and POM δ^15^N except for a few individual samples >7‰. Generally, plankton δ^15^N values did not differ between islands (*χ* ^2^ = 0.15, *p* = .696), but were affected by size fraction (*χ* ^2^ = 8.2, *p* = .016) in a stepwise pattern with the largest fraction (>300 μm) having higher δ^15^N (5.6% ± 1.2‰) than the two smaller fractions (*p* = .034, 153–300 μm, 4.3‰ ± 1.5‰; *p* = .0003, 65–153 μm: 3.5‰ ± 1.7‰) (Figure S11, Table S3). Further environmental resource characterization (plankton, POM) can be found in Appendix S1 (Table S3).

While there was a trend of negative mean δ^13^C_host‐symbiont_ (Δ^13^C) in Lizard corals (−0.5‰ ± 0.4‰) compared to positive mean δ^13^C_host‐symbiont_ in Heron corals (0.2‰ ± 1.1‰) before the heatwave, this was not significantly different (*χ* ^2^ = 1.78, *p* = .183) (Figure 5b, Table S5). At the holobiont level within Lizard Island, *P. verrucosa* associated with *C. pacificum* had lower δ^13^C_host‐symbiont_ (Δ^13^C) values in adults (−1.0‰ ± 0.4‰) than its juvenile counterparts (−0.35‰ ± 0.26‰) and similarly for *P. bairdi*–*C. pacificum* adults (−0.46‰ ± 0.11‰) versus juveniles (−0.18‰ ± 0.22‰) while the opposite pattern was observed for *S. pistillata*‐*Cladocopium* "C8 group" corals (Table S5). Interestingly, before the heatwave there was a relationship between host protein and δ^13^C_host‐symbiont_ (Δ^13^C), both proxies for heterotrophy, with negative δ^13^C_host‐symbiont_ values correlated with increasing protein concentrations for all holobiont pairs (*F*_1,83_ = 4.48, *p* = .037) (Figure S15a). For the only host‐Symbiodiniaceae holobiont present in both locations (*S. pistillata*–*Cladocopium "*C8 group"), the relationship between host protein and δ^13^C_host‐symbiont_ was more significant (*F*_1,44_ = 18.2, *p* = .0001) (Figure S15b).

### 3.7 Multivariate phenotypes reveal divergent response across space and time

**FIGURE 6:**
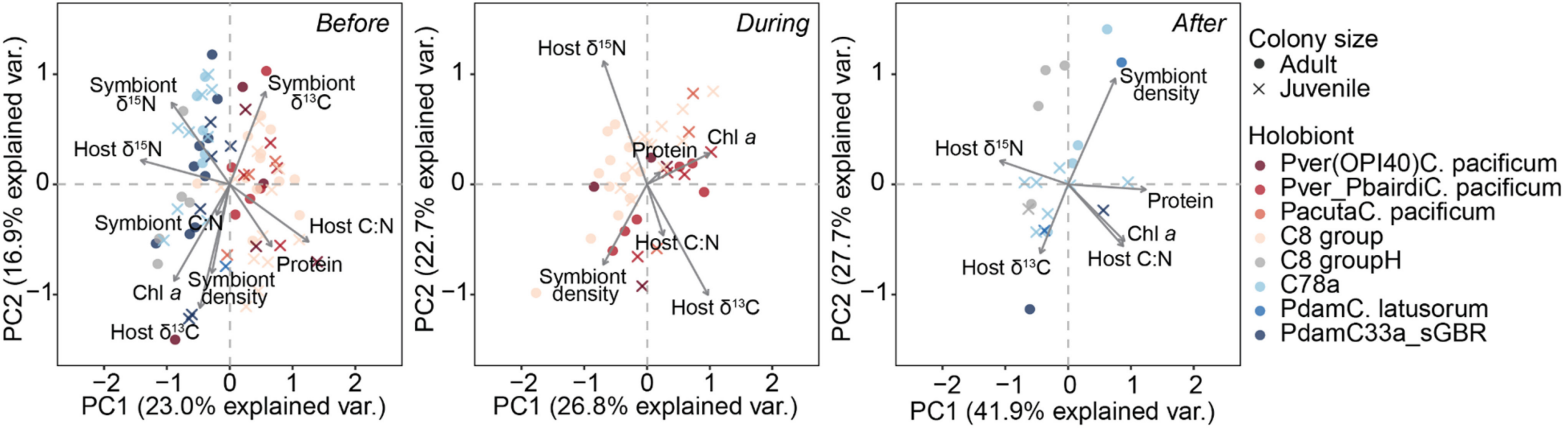
Principal component analysis (PCA) of coral multivariate phenotypes across the Great Barrier Reef before, during, and after the marine heatwave in 2020. Biplot vectors represent physiological parameters (Chl‐*a*, chlorophyll *a*).

PERMANOVA testing revealed a significant three‐way interaction among location, colony size, and time (pseudo *F* = 4.01, *p* = .01) as well as location, coral genus, and time (pseudo *F* = 2.8, *p* = .04). PCA further revealed distinct patterns through time. Before the heatwave, the first two principal component (PC) axes explained 23.0% and 16.9% of the variance, respectively (Figure 6). Significant separation was observed between locations, with Lizard Island corals grouping in neighboring space regardless of genus or colony size and most closely aligning with PC1 (e.g., host δ^13^C, host C:N ratio, and protein densities) (Figure 6). On the other hand, Heron Island corals, regardless of genus or colony size, grouped together, yet aligned with PC2 (e.g., symbiont δ^15^N, host δ^15^N, symbiont C:N ratio) (Figure 6).

During the heatwave, the first two axes explained 26.8% and 22.7% of the variance, respectively, where PC1 was most closely correlated with chlorophyll *a* and PC2 with host δ^13^C, host δ^15^N, and symbiont densities (Figure 6). Significant groupings emerged by colony size regardless of genus, with adults most closely aligned with symbiont density, host δ^13^C, and host δ^15^N and juveniles aligned with chlorophyll *a*. After the heatwave, the first two axes explained 41.9% and 27.7% of the variance, respectively. PC1 was most closely correlated with chlorophyll *a*, host C:N, protein, symbiont density and host δ^13^C, and PC2 with host δ^15^N (Figure 6). Significant groupings emerged by genus, with *Stylophora* most closely aligning with symbiont density, host δ^13^C, and host δ^15^N.

## 4 DISCUSSION

### 4.1 Recent exposure to marine heatwaves influences genotypic diversity

Fine‐scale genetic variability is known to influence patterns of bleaching susceptibility and mortality (Burgess et al., 2021; Sampayo et al., 2008; Wall et al., 2021). In this study, we discovered a diversity of pocilloporid host–Symbiodiniaceae associations, with only one shared partnership between the northern and southern GBR locations (*S. pistillata*–*Cladocopium* “C8 group”). Patterns in species composition may relate to adaptation to distinct thermal regimes over evolutionary time (Ulstrup et al., 2006). Alternatively, past exposure to marine heatwaves can lead to a shift to more stress‐tolerant symbionts through shuffling (Cunning et al., 2015) or the disappearance of weak corals or symbioses from the population through mortality (Burgess et al., 2021; Hughes, Kerry, et al., 2018; Pratchett et al., 2013; Quigley et al., 2022; Sampayo et al., 2008). Indeed, in our study, we found evidence that marine heatwaves can lead to less genetic diversity and potentially, the local disappearance of genotypes from the population. Specifically, at Lizard, *Stylophora* that experienced the 2016 and 2017 marine heatwaves were found to exclusively partner with the *Cladocopium* “C8 group,” and remarkably, not one transplant fragment died during the 2020 heatwave (100% survival). In contrast, at Heron, three associations were found within *Stylophora* that had not experienced the 2016 and 2017 heatwaves (*Cladocopium* "C8 group", "C35a", "C78a"). All corals possessing *Cladocopium* “C35a” died in the aftermath of the heatwave (0% survival) while corals with the “C8 group” (50% survival) or “C78a” symbionts (>75% survival) fared better, agreeing with an earlier study identifying these two symbionts as more thermally tolerant (Sampayo et al., 2008). Similarly for *Pocillopora*, corals with *Cladocopium* “C33a” did not survive the heatwave (<5% survival) and the small minority of corals harboring generalist *C. latusorum* (Turnham et al., 2021) appeared more resistant (>50% survival). At the coral host level, *P. bairdi*, *P. verrucosa*, and *P. acuta* represented our samples at Lizard (Schmidt‐Roach et al., 2013), whereas only *P. damicornis* was found in our samples at Heron. It is surprising that not a single colony of *P. damicornis* was found at Lizard, despite our extensive searches for corals containing its particular morphology. *Pocillopora damicornis* is described as locally persistent across the reef sites investigated and mentioned in >130 studies from Lizard Island between 1976 and 2018 (see ESM appendix 3 in Richards et al., 2021). The discrepancy with prior studies may be due to recent thermal stress, where *P. damicornis* may have been more sensitive to the 2016 and 2017 heatwaves, resulting in a significant reduction in the population or even local extinction (Tkachenko, 2015). This hypothesis is supported by our observation of the near complete mortality of transplant *P. damicornis* in the aftermath of the 2020 heatwave at Heron (~10% survival). Ultimately, regional differences in symbiont species associations uncovered here align with previous observations (Sampayo et al., 2007; Tonk et al., 2014) and are likely the result of evolutionary adaptation across a large spatial gradient, whereas the local disappearance of *P. damicornis* appears to be a result of ecological memory of recent severe disturbances, suggesting more thermally tolerant coral host–Symbiodiniaceae associations will be favored in a changing climate.

### 4.2 Ecological memory of marine heatwaves promotes bleaching resistance

Corals at Lizard Island, experiencing a third marine heatwave in 5 years, exhibited visual bleaching thresholds that were twice as high as those at Heron Island, which had experienced no significant coral bleaching in >10 years (Hughes et al., 2017; Quigley et al., 2022). These encouraging signatures of ecological memory corroborate findings from aerial bleaching surveys across the GBR in 2017 and 2020 (Hughes et al., 2019, 2021) and align with other studies from the Caribbean (Fisch et al., 2019; Gintert et al., 2018) to the Pacific (Guest et al., 2012; Pratchett et al., 2013), suggesting that some surviving corals are exhibiting higher bleaching thresholds in successive marine heatwaves. Interestingly, before the 2020 heatwave, both *Pocillopora* and *Stylophora* at Lizard were paler than conspecifics at Heron, with recent work suggesting corals with a lower symbiont load may be more bleaching‐resistant (Cornwell et al., 2021). These differential patterns in coral color can be explained by: (i) naturally divergent symbiont densities across distinct coral holobionts, where for example, *Stylophora* with *Cladocopium* “C8a” have been shown to have ~50% fewer symbionts than other holobionts at the same location (e.g., *Stylophora* associated with "C35a", or "C78a") (Kaniewska & Sampayo, 2022), (ii) unique environmental regimes between sites or locations (e.g., PAR) leading to adjusted symbiont densities or pigment concentrations (Anderson et al., 2017; Kaniewska & Sampayo, 2022), and/or (iii) incomplete recovery from recent marine heatwaves (Ritson‐Williams & Gates, 2020). At the reef level, bleaching probability and mortality has been shown to increase with greater colony size in *Pocillopora* (Burgess et al., 2021; Speare et al., 2022). Indeed, after the heatwave at Heron Island, comprehensive reef‐wide surveys demonstrated that mortality increased with greater colony size (Figure S8). During the heatwave at Lizard Island, however, we found the opposite pattern in bleaching susceptibility, suggesting larger *Pocillopora* colonies (≥25 cm) that survived the 2016 and 2017 heatwaves had greater resistance to the 2020 heatwave. Interestingly, *Stylophora* showed no correlation between bleaching prevalence and colony size at Lizard, possibly due to the absence of particularly large (≥25 cm) coral colonies from the population. Ultimately, across the fragment, colony, and reef levels, our results provide support of ecological memory promoting bleaching resistance across marine heatwaves, while also highlighting that the direction and effect of ecological memory can vary by species.

### 4.3 Maintenance or impairment of calcification rates in response to repeat heatwaves is species‐specific

Despite experiencing greater heat stress, *Pocillopora* at Lizard continued to calcify at the highest rates, suggesting that there was limited impairment of this energy‐demanding process during the heatwave. Importantly, the timing of measurements in our study differed due to travel restrictions associated with the COVID‐19 pandemic, which may have influenced the observed patterns. At Lizard, measurements occurred at the peak of the heatwave (March 2020). At Heron, measurements occurred 5 months after the heatwave (August 2020), at which time there was severe partial mortality in surviving *Pocillopora* corals (90%), leaving the majority of the skeleton exposed to increased chemical and biological erosion (Leggat et al., 2019). Although our study does not include post‐heatwave data from Lizard Island, coral cover increased following the 2020 marine heatwave (Tebbett et al., 2022), suggesting minimal bleaching‐related mortality occurred, whereas significant declines in coral cover were observed at Heron (Brown et al., 2023). In non‐heatwave years, calcification rates are greater at lower latitudes, which have been hypothesized to be a result of warmer seawater temperatures experienced throughout the year (Anderson et al., 2017). While this is indeed what we observed in *Pocillopora*, surprisingly the opposite was found for *Stylophora*, with greater calcification rates at higher latitudes, despite the aforementioned timeframe discrepancies and greater partial mortality (~5% in *Stylophora* at Lizard vs. >60% at Heron). This may be due to legacy effects of the 2016 and 2017 marine heatwaves, with previous studies showing decreased growth rates following thermal stress (Baumann et al., 2019; Cantin & Lough, 2014) or alternatively, species‐specific patterns in symbiont associations, where associations with more thermally tolerant symbionts can lead to lower growth rates (Cunning et al., 2015; Jones & Berkelmans, 2010). Although small sample sizes prevented robust statistical analyses, in the limited specimens that had the same host–symbiont association across locations (*S. pistillata*–*Cladocopium* “C8 group”), adult corals at Heron seemingly had greater calcification rates than Lizard, lending support to the idea that physiological damage may accumulate across marine heatwaves in the absence of a sufficient recovery period (i.e., sensitization). Indeed, we found further support of sensitization within locations, where adult *Stylophora* experiencing the third marine heatwave in 5 years at Lizard had reduced calcification rates when compared to “naïve” juveniles. However, we are unable to separate the history of disturbance from colony age, with earlier work suggesting young fragments may calcify faster than old fragments (Dornelas et al., 2017; Elahi & Edmunds, 2007). At the holobiont level (i.e., the same species), locations with and without a recent history of disturbance demonstrated higher calcification rates in some juveniles (e.g., *P. verrucosa*, *S. pistillata*‐“C78a”), but not all (e.g., *P. bairdi*). Therefore, it remains inconclusive whether the differential patterns in calcification observed here are a direct result of memory of recent severe disturbances or age, and support for either hypothesis in the literature is limited. Nevertheless, the results presented here build support for the hypothesis that prior exposure to marine heatwaves may impair calcification rates in some species but not in others.

### 4.4 Trophic plasticity enhances organismal performance in subsequent marine heatwaves

Generally, fed corals have higher protein content relative to unfed corals (Borell et al., 2008; Hoogenboom et al., 2015; Rangel et al., 2019; Tremblay et al., 2014) and fed corals can maintain higher protein content during heat stress and recovery (Connolly et al., 2012; Ezzat et al., 2016). In our study, we found evidence that marine heatwaves can lead to greater levels of host protein, which become elevated in surviving corals during/after heat stress, potentially as a result of increased heterotrophy and/or differences in resource acquisition. Adult *Pocillopora* and *Stylophora* that had experienced the 2016 and 2017 heatwaves at Lizard Island had distinct (non‐overlapping) coral host isotopic niches and also significantly greater protein content than juvenile conspecifics that had not experienced back to back bleaching, which may be a result of heterotrophic feeding as observed in experimental work (Ferrier‐Pagès et al., 2003; Krueger et al., 2020). Differential trophic strategies may be related to: (i) the history of environmental stress (Hughes & Grottoli, 2013; Radice et al., 2022; Schoepf et al., 2015), (ii) inherent species‐specific biology (Conti‐Jerpe et al., 2020; Grottoli et al., 2006), and/or (iii) differences in nutritional resources between regions (Fox et al., 2018). Large plankton δ^15^N values (>300 μm) were similar in both locations (before timepoint) indicating similar access to at least some oceanic resources while variability in smaller size fractions may represent a mixture of lagoonal and oceanic nutrient subsidies. However, Heron Island had higher mean δ^15^N values for a non‐symbiotic coral (7.3‰) and both coral host genera (4.6‰) compared to Lizard Island coral hosts (3.7‰), with the former values more similar to δ^15^N–NO_3_^−^ (6.3‰) of upwelled deep ocean water (Coral Sea; Yoshikawa et al., 2015), indicating that Heron Island reefs may receive deep oceanic nutrients via the Capricorn Eddy (Weeks et al., 2010).

Within the same genus and location, *Pocillopora* that had experienced the 2016 and 2017 heatwaves (i.e., adults at Lizard) had greater protein content and a trend of higher host δ^15^N values (4.1‰) than naïve conspecifics (i.e., juvenile = 3.7‰), agreeing with similar increases in experimentally fed corals (Hoogenboom et al., 2015). Interestingly, across all host‐Symbiodiniaceae holobionts, increasing protein concentrations were correlated with negative δ^13^C_host‐symbiont_ (Δ^13^C) values that are often used as a proxy for heterotrophy (Ferrier‐Pagès et al., 2011; Fox et al., 2018). Notably, there was a stronger relationship between protein and δ^13^C_host‐symbiont_ for the one host‐Symbiodiniaceae holobiont present in both locations (*S. pistillata*–*Cladocopium* “C8 group”), demonstrating the robust relationship across all host–Symbiodiniaceae holobionts despite inherent biological variability and environmental impacts. Changes in biochemical composition of tissue biomass (e.g., protein) may relate to changes in δ^13^C values (Hayes, 2018). Following this reasoning, corals that had experienced the 2016 and 2017 heatwaves were more heterotrophic than corals that had not. This suggests that a loss of symbionts and associated reduction in autotrophy (e.g., lower %C in corals that experienced 2016 and 2017 heatwaves) due to bleaching may force increased heterotrophy to ensure survival (Dobson et al., 2021)—a hypothesis that is further supported by our observation of increases in protein content during/after bleaching in *Pocillopora*. Lizard corals of both genera increased host %C and chlorophyll *a* during bleaching, suggesting heterotrophy had a positive feedback loop that stimulated symbiont autotrophy (Lyndby et al., 2019; Tremblay et al., 2014, 2016). Whether the increase in host %C in Lizard corals is due to gaining carbon via heterotrophy (Baumann et al., 2014; Tremblay et al., 2016) or due to the release of carbon from energy stores such as lipids (Axworthy et al., 2022) cannot be determined, and is likely species‐specific. However, for Lizard Island *Stylophora* specifically, increased host %C accompanied by increased host δ^13^C values suggests catabolism of carbon‐rich lipids that left remaining tissue ^13^C‐enriched (Grottoli et al., 2004, 2017; Wall et al., 2019), but this does not preclude concurrent heterotrophic feeding. The change in host δ^13^C of Lizard Island *Stylophora* from before to during bleaching was similar in adults (−16.0‰ to −14.8‰) and juveniles (−16.7‰ to −15.5‰). Compared to Lizard Island *Stylophora* host δ^13^C during bleaching (−15.2‰), similar host δ^13^C values were observed during a past winter (−15.4‰, June 2008; Blanckaert et al., 2020). This suggests the *Stylophora* population at Lizard was physiologically plastic, exhibiting changes in biochemical composition and subsequently tissue stable isotopes due to environmental stress (Wall et al., 2019). Taken together, trophic plasticity may beneficially influence organismal performance to subsequent marine heatwaves.

## 5 CONCLUSIONS

Coral reefs are in serious danger, with accelerating ocean warming currently considered the greatest threat to reef survival (Hughes, Kerry, et al., 2018). This study makes the critically important step of identifying that history of severe coral bleaching in 2016 and 2017 equates to stress tolerance in a successive heatwave through physiological legacies and shifts in community composition. Concerningly, increased bleaching resistance was partially attributable to a loss of genetic diversity, with the local disappearance of *P. damicornis* uncovered in the northern GBR at Lizard Island, underscoring that modern heatwaves threaten biodiversity (Starko et al., 2023). More encouragingly, surviving corals experiencing the third marine heatwave in 5 years were two times less sensitive to coral bleaching than conspecifics without ecological memory. Further, a history of heat stress was found to modulate trophic strategies, where a greater reliance on heterotrophy in corals with ecological memory rendered corals more successful (i.e., greater survival) than corals with greater reliance on symbiont autotrophy. Yet, divergent species‐specific responses in calcification rates highlight the significant role of previous marine heatwaves influencing reef growth and accretion. On one hand, *Pocillopora* that had survived the 2016 and 2017 marine heatwaves (Lizard) were able to maintain three times greater calcification rates than conspecifics at Heron, whereas on the other hand, adult *Stylophora* calcification was lower relative to naïve juvenile conspecifics as well as adults and juveniles at Heron, contrasting earlier studies that demonstrate increasing growth rates at lower latitudes (e.g., Anderson et al., 2017). Together, the results of this study demonstrate that the response of corals to subsequent thermal stress events is contingent on previous events through the selection for the most resilient genotypes (i.e., those that are physiologically plastic). While beneficial acclimatization is apparent in some species, trade‐offs to survival exist in others, highlighting the complexities of predicting coral performance and resilience in the Anthropocene.

## AUTHOR CONTRIBUTIONS

**Kristen T. Brown:** Conceptualization (lead); data curation (lead); formal analysis (lead); funding acquisition (lead); investigation (lead); methodology (lead); project administration (lead); resources (lead); software (lead); supervision (lead); visualization (lead); writing – original draft (lead); writing – review and editing (lead). **Amatzia Genin:** Conceptualization (lead); data curation (supporting); formal analysis (supporting); investigation (supporting); methodology (supporting); supervision (lead); writing – review and editing (supporting). **Matheus A. Mello‐Athayde:** Conceptualization (lead); data curation (supporting); investigation (supporting); methodology (supporting); resources (supporting). **Ellie Bergstrom:** Formal analysis (supporting); methodology (supporting); writing – review and editing (supporting). **Adriana Campili:** Formal analysis (supporting); investigation (supporting); methodology (supporting). **Aaron Chai:** Formal analysis (supporting); investigation (supporting); methodology (supporting). **Sophie G. Dove:** Conceptualization (supporting); formal analysis (supporting); funding acquisition (supporting); methodology (supporting); resources (supporting); writing – review and editing (supporting). **Maureen Ho:** Formal analysis (supporting); investigation (supporting); writing – review and editing (supporting). **Devin Rowell:** Formal analysis (supporting); investigation (supporting). **Eugenia M. Sampayo:** Data curation (supporting); formal analysis (supporting); investigation (supporting); methodology (supporting); resources (supporting); visualization (supporting); writing – original draft (supporting); writing – review and editing (supporting). **Veronica Z. Radice:** Conceptualization (lead); data curation (lead); formal analysis (lead); funding acquisition (lead); investigation (lead); methodology (lead); project administration (lead); resources (lead); software (lead); supervision (lead); visualization (lead); writing – original draft (lead); writing – review and editing (lead).

## Supporting information


Appendix S1.

## Data Availability

Original data and all R‐scripts generated for this study can be found on https://github.com/imkristenbrown/Coral‐ecological‐memory‐from‐heatwaves. Sequence data were deposited on GenBank under accession numbers OR887551 ‐ OR887595 and OR837528 ‐ OR837623.
