# Supplementary material for: Marine heatwaves modulate the genotypic and physiological responses of reef‐building corals to subsequent heat stress
Source: Ecol Evol. 2023 Dec 13;13(12):e10798. doi: 10.1002/ece3.10798 (PMC10719612; doi:10.1002/ece3.10798)
Supplement: Supplementary file 1 — Appendix S1. [file ECE3-13-e10798-s001.pdf]

Supplemental Material to:

Marine heatwaves modulate the genotypic and physiological responses of reef-building corals to subsequent heat stress

Kristen T. Brown*, Amatzia Genin, Matheus A. Mello-Athayde, Ellie Bergstrom, Adriana Campili, Aaron Chai, Sophie G. Dove, Maureen Ho, Devin Rowell, Eugenia M. Sampayo, and Veronica Z. Radice

*Corresponding author; kristen.brown@uq.edu.au

Materials and Methods

Site selection, environmental records and benthic community characterization

Before the heatwave (November/December 2019), benthic community composition was determined along 8 × 30 m transects per site using the modified methodology of (Bryant et al., 2017). Quadrats (0.25 m^2) were placed alternately left and right along 30 m transect tapes every 0.5 m, totaling 30 photo quadrats per transect and 720 per location (8 transects, 30 quadrats per transect, 3 sites). Benthic community composition was measured through semi-automated analysis via CoralNet using 30 randomly allocated points per photo quadrat (Bryant et al., 2017) from 22 benthic categories, with the four central substrate categories consisting of hard coral, other invertebrates, macroalgae, and abiotic substrate (Figure 1, Table S1). A total of 122 and 143 photo quadrats were manually annotated per location (Heron and Lizard, respectively) until at least 80% accuracy was obtained, at which point the remaining images were classified via machine learning.

In order to assess the isotope end members of planktonic food sources, plankton and particulate organic matter (POM) were sampled at each site. Daytime (10:00–15:00) plankton tows and seawater collections were made at each site (n = 3) using 10 minute subsurface (1-2 m depth) net tows (speed ~0.5 m/s), with: (i) a 153 µm net (29 cm diameter) for large fractions, and either (ii) a 53 µm net (30 cm diameter) or a 65 µm hand net (21 cm diameter) for the

smaller fraction (65–153 μm). In the lab, the plankton were fractionated by size into three fractions: 65–153 μm, 153–300 μm, and >300 μm (n = 10–13 per fraction). Seawater (15L) was collected in thoroughly-rinsed polystyrene bottles and pre-filtered through 65 μm mesh, then vacuum-filtered onto pre-combusted glass fiber filters (0.7 μm nominal pore size; Whatman GF/F) to collect POM (n=27 Lizard, n=32 Heron).

Focal species size demographics and bleaching severity assessments

Two widespread and common coral genera, *Pocillopora* and *Stylophora*, were selected to gather field measurements of individual colonies before, during and after the 2020 thermal stress event. The size demographics of *Pocillopora* and *Stylophora* between the two study locations was determined before the 2020 heatwave by recording the size of each *Pocillopora* or *Stylophora* colony encountered within a 1-m belt transect along the 30 m transects used to determine community composition (as above; 0.5 m on either side of the transect tapes; Figure S2).The diameter (cm) of each colony was categorized into six discrete groups (<10, 11–20, 21–30, 31–40, 41–50, >51).

During and after the heatwave in March 2020 and August 2020 at Lizard and Heron, respectively, coral colony color of the two focal genera was assessed as an indicator of bleaching severity. Each colony of *Pocillopora* and *Stylophora* encountered along the 1m belt transects (n = 4–8 per site) was measured (to the nearest cm) and its color recorded *in situ* in relation to standardized color cards (Siebeck et al., 2006). Due to observations of recent mortality at Heron, colonies were also scored for partial mortality (%).

Genetics

A small chip (2–3 mm) of each fragment was used to extract gDNA using a MoBio DNeasy Powersoil DNA extraction kit (cf. manufacturer's instructions). Coral species identification for *Pocillopora* was confirmed with a genetic assay using the mitochondrial ORF region (Schmidt-Roach et al., 2013). The extracted DNA was diluted 1:20 and PCR was done using the FATP/RORF primers (cf. (Flot & Tillier, 2007)) and NEB OneTAQ Mastermix under cycling conditions: 95°C 2 min initial denaturation, 35 cycles of 94°C 30s, 53°C 30s, 68°C 1 min, and a final extension of 68°C for 10 min. All amplicons were cleaned using ExoSap-IT and Sanger sequenced in a single direction using the forward FATP primer at the Australian Genome Research Facility (Australian Genome Research Facility (AGRF), Brisbane, Australia). The species of resident coral endosymbionts (Symbiodiniaceae) in both *Pocillopora* and *Stylophora* were identified using PCR amplification of the ITS2 rDNA region followed by denaturing gradient electrophoresis alongside local ‘library’ reference specimens with confirmed symbiont identifications (Sampayo et al., 2009). To cross-confirm, dominant bands of characteristic profiles were excised, re-amplified and sequenced using the ITS2intfor and ITS2rev primers (cf. (LaJeunesse et al., 2003; Sampayo et al., 2009)). For the *Pocillopora* specimens only, the chloroplast minicircle psbA non-coding and partial coding region was amplified using the psbAFor-1/psbARev-1 primers (LaJeunesse & Thornhill, 2011) to characterize samples against two recently identified *Pocillopora* associated symbiont species, *Cladocopium latusorum* and *Cladocopium pacifium* (Turnham et al., 2021). Successful PCR amplicons were cleaned using ExoSap-IT and bi-directionally Sanger sequenced (AGRF, Brisbane, Australia). All sequence chromatograms (host mtORF; symbiont ITS2 rDNA and chloroplast psbA) were visually inspected and compared to holotype sequences from described *Pocillopora* species (Schmidt-Roach et al., 2013, 2014), symbiont ITS2-types (*Pocillopora* and *Stylophora* -(Sampayo et al., 2009)) and *Cladocopium* species (*Pocillopora* -(Turnham et al., 2021)). Sequences were aligned (MAFFT) and adjusted by eye, representing a quality control process

that was particularly important given the large number of indels present in the psbA sequence dataset. For the ORF sequences a phylogeny was constructed based on a maximum parsimony heuristic search. All indels were adjusted to represent a single base change, using gaps as a 5th character state. A majority rule consensus tree was produced with bootstrap values calculated (TBR branch swapping, random addition) from 1000 replicates in PAUP (vs. 4.0a, build 169; (Swofford & Others, 2002). Since the psbA (partial coding, and entire non-coding region) contained a large amount of indels, a maximum likelihood phylogeny was constructed (does not account for gaps) using a heuristic search with bootstrap values calculated (1000 replicates) using IQtree. Sequences were submitted to GenBank under accession numbers OR837528–OR837623 and OR887551–OR887595.

Results

Symbiont carbon and nitrogen elements and stable isotopes. Symbiont data was obtained from both regions only from the before timepoint (pre-bleaching, 2019) due to insufficient material post-bleaching. Symbiont $\delta^{15}N$ was affected by the interaction between genus and colony size ($\chi2$= 4.38, *p*=0.036) (Table S3). *Stylophora* juveniles had higher symbiont $\delta^{15}N$ values (3.3‰) compared to *Pocillopora* juveniles (2.5‰, p=0.001). Juvenile *Pocillopora* had lower symbiont $\delta^{15}N$ (2.5‰) compared to *Pocillopora* adults (3.1‰, p=0.042) and *Stylophora* adults (3.3‰, p=0.003). Symbiont $\delta^{13}C$ was affected by location ($\chi2$ = 4.42, p=0.035) due to higher symbiont mean $\delta^{13}C$ at Lizard (-15.8 ± 0.8‰) compared to Heron Island (-16.8 ± 1.3‰).

Region affected symbiont %C ($\chi2$ = 8.19, , *p*=0.004), which was higher at Heron (41.8%) compared to Lizard island (22.5%). Symbiont C:N was affected by region and genus ($\chi2$ = 8.34, *p*=0.004), with Heron *Pocillopora* (7.7) having higher C:N than Heron *Stylophora* (6.7) (*p*=0.001). Symbiont C:N was also affected by colony size ($\chi2$ = 4.28, *p*=0.038) due to slightly

higher C:N (7.1) in *Pocillopora* compared to juveniles (6.8) although the pairwise contrast was not significant (p=0.166).

Coral trophic ecology between regions. Plankton, POM, and *Tubastraea* cf. *coccinea* (non-symbiotic, scleractinian coral) data were obtained from both regions only before the bleaching (2019) and plankton/POM only from Lizard Island during the bleaching (2020). POM $\delta^{15}N$ differed significantly by location ($\chi 2$ = 17.5, p<0.0001) with higher POM $\delta^{15}N$ at Lizard Island (5.3 ± 1.4‰) compared to Heron (3.5 ± 1.5‰) (Figure S11, Figure S14, Table S3). Similarly, location affected POM $\delta^{13}C$ ($\chi 2$ = 10.0, p= 0.002), which was lower at Heron (-23.7 ± 2.0‰) compared to Lizard Island (-20.9 ± 3.1‰). Coral isotopic niches at Heron Island overlapped with the smallest plankton fraction (65–153 µm) although this size fraction had highly variable $\delta^{13}C$ values (Figure S14). At Heron Island some POM samples were dominated by *Trichodesmium* blooms following calm conditions in the preceding weeks (observed water surface slicks and confirmation of *Trichodesmium* bundles under microscope).

Plankton $\delta^{13}C$ was influenced by the interaction between location and size fraction ($\chi 2$ = 6.5, p=0.039), where at Heron, the smallest fraction (65–153 µm) had the highest $\delta^{13}C$ (-17.8 ± 2.6‰) compared to the 153–300 µm fraction with the lowest $\delta^{13}C$ (-20.6 ± 0.9‰; p=0.0001) and the >300 µm fraction (-20.4 ± 0.6‰; p=0.0003) (Figure S14, Table S3). Before the heatwave, host and symbiont isotopic niches did not overlap for either *Pocillopora* or *Stylophora* in either location (Figure S14).

Host C:N ratio was affected by time ($\chi 2$ = 14.761, p < 0.001), the interaction between location and coral genus ($\chi 2$ = 5.903, p = 0.015), and the interaction between location and treatment ($\chi 2$ = 3.929, p = 0.047) (Figure 3h, Table S3). Pairwise comparisons showed that at Heron Island, host C:N (mean ± SD) was slightly higher in *Pocillopora* (5.41 ± 0.23) compared to *Stylophora*

(5.30 ± 0.21; $p=0.006$), while Lizard Island *Stylophora* host C:N (5.54 ± 0.25) was slightly higher than conspecifics at Heron ($p=0.023$). Further, juvenile coral host C:N was slightly higher at Lizard Island (5.62 ± 0.28) than Heron (5.36 ± 0.23; $p=0.044$). Overall, host C:N slightly increased from before (5.42 ± 0.28) to during / after (5.54 ± 0.28) the marine heatwave ($p<0.0001$).

Supplemental tables

Table S1. Benthic community composition was determined from photoquadrats via CoralNet from 22 benthic categories.

Benthic category	CoralNet short code	Functional group
Acroporidae (branching)	ACR_br	Hard coral
Acroporidae (table/coryombose/digitate)	ACR_t_c_d	
Acroporidae (plate/encrusting)	ACR_p_e	
Pocillopora	Pocill	
Stylophora	Styloph	
Poritidae (massive)	POR-MASS	
Diploastrea	Diplo	
Favidae-Mussidae (massive/meandroid)	FAV-MUS	
Other hard coral	OTH-HC	
Other sessile invertebrates	OTH-SINV	Other invertebrates
Mobile invertebrates	mob	
Sponge	Sponge	
Soft coral	Soft	
Padina	Pad	Algae
Halimeda	Halimeda	
Crustose coralline algae (CCA)	CCA	
Turf	Turf	
Other fleshy algae	Alg_fleshy	
Broken coral rubble	C-Rubble	Other
Sand	Sand	Soft substrate
Dead coral	D_coral	Hard substrate
Bare substrate	Bare-Subst	

**Table S2**. The University of Queensland Stable Isotope Geochemistry Laboratory standards for isotope analyses.			
**Reference ID**	**Name**	**$\delta^{13}C$ (‰)**	**$\delta^{15}N$ (‰)**
USGS-40	L-glutamic acid	-26.39	-4.52
USGS-41a	L-glutamic acid enriched in 13C and 15N	36.55	47.55
USGS-43	Indian human hair powder (<60 mesh)	-21.28	8.44

Table S3. Summary of coral host, symbiont, non-symbiotic coral, plankton, and particulate organic matter (POM) carbon and nitrogen stable isotopes and concentrations (mean ± SD) and C:N ratios from Heron (southern GBR) and Lizard (northern GBR) Islands from Before (November-December 2019), During (February 2020), and After (August 2020) the 2020 marine heatwave that caused mass coral bleaching event across the Great Barrier Reef, Australia.

Reef	Fraction	Genus	Colony size	Time	n	$\delta^{13}C$ (‰)	$\delta^{15}N$ (‰)	%C	%N	C:N molar
Heron	Coral Host	*Pocillopora*	Adult	Before	12	-17.0 ± 1.0	4.5 ± 0.3	16.1 ± 2.5	3.6 ± 0.6	5.3 ± 0.1
				After	5	-16.1 ± 1.3	5.0 ± 0.3	8.9 ± 2.9	1.8 ± 0.7	5.7 ± 0.3
			Juvenile	Before	12	-16.3 ± 0.9	4.5 ± 0.5	15.1 ± 2.3	3.3 ± 0.5	5.4 ± 0.1
				After	4	-16.2 ± 0.7	4.6 ± 0.3	9.9 ± 6.0	2.1 ± 1.3	5.6 ± 0.2
		Stylophora	Adult	Before	12	-16.4 ± 0.8	4.7 ± 0.4	13.0 ± 2.3	2.9 ± 0.5	5.2 ± 0.1
				After	11	-16.2 ± 0.6	4.9 ± 0.5	9.1 ± 3.7	2 ± 0.8	5.4 ± 0.1
			Juvenile	Before	12	-16.4 ± 0.8	4.7 ± 0.4	10.1 ± 2.5	2.3 ± 0.6	5.2 ± 0.1
				After	10	-16.6 ± 0.7	4.6 ± 0.5	6.9 ± 3.5	1.5 ± 0.8	5.4 ± 0.3
Lizard		*Pocillopora*	Adult	Before	12	-16.1 ± 1.0	4.1 ± 0.2	10.9 ± 2.7	2.4 ± 0.6	5.3 ± 0.2
				During	8	-15.7 ± 0.6	4.0 ± 0.2	17.0 ± 3.2	3.4 ± 0.6	5.8 ± 0.2
			Juvenile	Before	12	-16.3 ± 0.7	3.7 ± 0.4	7.9 ± 2.9	1.6 ± 0.7	5.7 ± 0.3
				During	10	-16.4 ± 1.4	3.7 ± 0.3	16.5 ± 2.6	3.4 ± 0.5	5.7 ± 0.2
		Stylophora	Adult	Before	12	-16.0 ± 0.3	3.7 ± 0.5	5.9 ± 1.5	1.2 ± 0.3	5.6 ± 0.2
				During	12	-14.8 ± 0.3	3.9 ± 0.3	10.3 ± 3.0	2.2 ± 0.7	5.5 ± 0.3
			Juvenile	Before	12	-16.7 ± 0.7	3.5 ± 0.3	5.0 ± 2.5	1 ± 0.5	5.7 ± 0.3
				During	12	-15.5 ± 0.5	3.8 ± 0.2	11.1 ± 2.5	2.4 ± 0.5	5.4 ± 0.2
Heron	Symbiont	*Pocillopora*	Adult	Before	11	-17.0 ± 2.0	3.2 ± 0.6	41.7 ± 7.4	6.2 ± 1.5	8.0 ± 1.2
			Juvenile	Before	9	-17.1 ± 1.0	2.5 ± 1.1	40.5 ± 20.1	6.6 ± 3.5	7.3 ± 0.8
		Stylophora	Adult	Before	11	-16.6 ± 0.9	3.7 ± 0.3	41.0 ± 10.0	7.3 ± 1.8	6.6 ± 0.5
			Juvenile	Before	12	-16.5 ± 0.8	3.8 ± 0.5	43.5 ± 10.7	7.6 ± 2.2	6.8 ± 0.6
Lizard		*Pocillopora*	Adult	Before	8	-15.6 ± 1.0	3.0 ± 0.4	26.8 ± 7.3	4.5 ± 1.2	6.9 ± 0.3

			Juvenile	Before	11	-16.1 ± 0.5	2.6 ± 1.1	22.2 ± 14.2	4.3 ± 3.2	6.1 ± 0.8
		Stylophora	Adult	Before	12	-15.6 ± 0.6	2.9 ± 0.5	22.0 ± 6.4	3.8 ± 1.2	6.8 ± 1.0
			Juvenile	Before	12	-16.0 ± 0.9	2.8 ± 0.5	20.5 ± 6.3	3.4 ± 1.1	7.1 ± 1.1
Heron	Non-symbiotic Coral Host	*Tubastraea*	N/A		5	-20.3 ± 0.7	7.3 ± 1.0	13.8 ± 3.0	3.3 ± 0.8	4.9 ± 0.4
	Plankton, >300 µm				10	-20.4 ± 0.6	5.5 ± 1.7	22.7 ± 5.6	5.4 ± 1.4	5.0 ± 0.3
	Plankton, 153-300 µm				10	-20.6 ± 0.9	3.8 ± 1.9	20.8 ± 8.5	4.7 ± 2.1	5.2 ± 0.3
	Plankton, 65-153 µm				10	-17.8 ± 2.6	4.0 ± 2.1	20.8 ± 6.7	3.7 ± 1.9	6.4 ± 1.0
	POM				32	-23.7 ± 2.0	3.5 ± 1.5	32.7 ± 7.7	4.8 ± 1.4	-
Lizard	Non-symbiotic Coral Host	*Tubastraea*			5	-21.2 ± 0	5.6 ± 0.5	16.2 ± 5.4	4.4 ± 1.4	4.3 ± 0.1
	Plankton, >300 µm				13	-19.7 ± 0.7	5.7 ± 0.6	28.4 ± 6.1	7.2 ± 2.0	4.8 ± 0.9
	Plankton, 153-300 µm				12	-20.0 ± 1.5	4.7 ± 1.0	18.7 ± 4.7	4.2 ± 1.2	5.3 ± 0.4
	Plankton, 65-153 µm				10	-19.1 ± 1.3	3.0 ± 1.3	11.7 ± 3.5	2.2 ± 0.8	6.0 ± 0.5
	POM				27	-20.9 ± 3.1	5.3 ± 1.4	-	-	-
Lizard	Plankton, >300 µm		N/A	During	7	-19.8 ± 0.6	4.9 ± 0.5	38.9 ± 5.5	7.7 ± 0.8	5.1 ± 0.4
	Plankton, 153-300 µm				6	-19.7 ± 0.6	4.6 ± 0.4	31.8 ± 7.9	6.4 ± 1.7	5.0 ± 0.2
	Plankton, 65-153 µm				5	-17.4 ± 3.6	4.2 ± 0.2	23.5 ± 7.0	3.7 ±1.5	6.7 ± 1.2

	POM				36	-20.5 ± 1.5	1.9 ± 1.3	-	-	-

Table S4. Coral and symbiont designations.

Sample ID	Collection date	Loca-tion	Site	Coral genus	Coral species	Symbiont ITS2 type	Symbiont species	psba seq genus	psba seq species
HA01	2019-12-04	Heron	Harry's Bommie	*Pocillopora*	*damicornis*	C33a*	C33a		
HA02	2019-12-04	Heron	Harry's Bommie	*Pocillopora*	*damicornis*	C33a*	C33a	*Symbiodinium*	*Symbiodinium sp.*
HA03	2019-12-04	Heron	Harry's Bommie	*Pocillopora*	*damicornis*	C33a*	C33a		
HA04	2019-12-04	Heron	Harry's Bommie	*Pocillopora*	*damicornis*	C33a*	C33a	*Symbiodinium*	*Symbiodinium sp.*
HA05	2019-12-04	Heron	Harry's Bommie	*Pocillopora*	*damicornis*	C33a*	C33a	*Symbiodinium*	*Symbiodinium sp.*
HA06	2019-12-04	Heron	Harry's Bommie	*Pocillopora*	*damicornis*	C33a	C33a	*Symbiodinium*	*Symbiodinium sp.*
HA07	2019-12-04	Heron	Harry's Bommie	*Pocillopora*	*damicornis*	C33a	C33a	*Symbiodinium*	*Symbiodinium sp.*
HA08	2019-12-04	Heron	Harry's Bommie	*Pocillopora*	*damicornis*	C33a*	C33a	*Symbiodinium*	*Symbiodinium sp.*
HA17	2019-12-08	Heron	Coral Canyons	*Pocillopora*	*damicornis*	C33a*	C33a	*Symbiodinium*	*Symbiodinium sp.*

HA18	2019-12-08	Heron	Coral Canyons	*Pocillopora*	*damicornis*	C33a*	C33a	*Symbiodinium*	*Symbiodinium sp.*
HA19	2019-12-08	Heron	Coral Canyons	*Pocillopora*	*damicornis*	C33a	C33a		
HA20	2019-12-08	Heron	Coral Canyons	*Pocillopora*	*damicornis*	C33a*	C33a	*Symbiodinium*	*Symbiodinium sp.*
HA21	2019-12-08	Heron	Coral Canyons	*Pocillopora*	*damicornis*	C33a*	C33a		
HA22	2019-12-08	Heron	Coral Canyons	*Pocillopora*	*damicornis*	C33a*	C33a	*Symbiodinium*	*Symbiodinium sp.*
HA23	2019-12-08	Heron	Coral Canyons	*Pocillopora*	*damicornis*	C33a*	C33a		
HA24	2019-12-08	Heron	Coral Canyons	*Pocillopora*	*damicornis*	C1b-c-42 a	*C. latusorum*	*Cladocopium*	*Cladocopium latusorum*
HA33	2019-12-11	Heron	Pam's Point	*Pocillopora*	*damicornis*	C33a*	C33a	*Symbiodinium*	*Symbiodinium sp.*
HA34	2019-12-11	Heron	Pam's Point	*Pocillopora*	*damicornis*	C33a*	C33a		
HA35	2019-12-11	Heron	Pam's Point	*Pocillopora*	*damicornis*	C1b-c-42 a	*C. latusorum*		
HA36	2019-12-11	Heron	Pam's Point	*Pocillopora*	*damicornis*	C33a*	C33a		
HA37	2019-12-11	Heron	Pam's Point	*Pocillopora*	*damicornis*	C1b-c-j-4 2a	*C. latusorum*	*Cladocopium*	*Cladocopium latusorum*
HA38	2019-12-11	Heron	Pam's	*Pocillopora*	*damicornis*	C33a	C33a	*Symbiodinium*	*Symbiodinium*

			Point						*sp.*
HA39	2019-12-11	Heron	Pam's Point	*Pocillopora*	*damicornis*	C33a	C33a	*Symbiodinium*	*Symbiodinium sp.*
HA40	2019-12-11	Heron	Pam's Point	*Pocillopora*	*damicornis*	C33a*	C33a		
HA49	2019-12-05	Heron	Harry's Bommie	*Stylophora*	*pistillata*	C78a	C78a		
HA50	2019-12-05	Heron	Harry's Bommie	*Stylophora*	*pistillata*	C78a	C78a		
HA51	2019-12-05	Heron	Harry's Bommie	*Stylophora*	*pistillata*	C78a	C78a		
HA52	2019-12-05	Heron	Harry's Bommie	*Stylophora*	*pistillata*	C78a	C78a		
HA53	2019-12-05	Heron	Harry's Bommie	*Stylophora*	*pistillata*	C35a	C35a		
HA54	2019-12-05	Heron	Harry's Bommie	*Stylophora*	*pistillata*	C78a	C78a		
HA55	2019-12-05	Heron	Harry's Bommie	*Stylophora*	*pistillata*	C78a	C78a		
HA56	2019-12-05	Heron	Harry's Bommie	*Stylophora*	*pistillata*				
HA65	2019-12-10	Heron	Coral Canyons	*Stylophora*	*pistillata*	C78a	C78a		
HA66	2019-12-10	Heron	Coral Canyons	*Stylophora*	*pistillata*	C78a	C78a		

HA67	2019-12-10	Heron	Coral Canyons	*Stylophora*	*pistillata*	C8a	C8 group		
HA68	2019-12-10	Heron	Coral Canyons	*Stylophora*	*pistillata*	C8a	C8 group		
HA69	2019-12-10	Heron	Coral Canyons	*Stylophora*	*pistillata*	C35a+	C35a		
HA70	2019-12-10	Heron	Coral Canyons	*Stylophora*	*pistillata*	C35a	C35a		
HA71	2019-12-10	Heron	Coral Canyons	*Stylophora*	*pistillata*	C78a	C78a		
HA72	2019-12-10	Heron	Coral Canyons	*Stylophora*	*pistillata*				
HA81	2019-12-12	Heron	Pam's Point	*Stylophora*	*pistillata*	C8a	C8 group		
HA82	2019-12-12	Heron	Pam's Point	*Stylophora*	*pistillata*	C8a	C8 group		
HA83	2019-12-12	Heron	Pam's Point	*Stylophora*	*pistillata*	C8a	C8 group		
HA84	2019-12-12	Heron	Pam's Point	*Stylophora*	*pistillata*	C8a	C8 group		
HA85	2019-12-12	Heron	Pam's Point	*Stylophora*	*pistillata*	C8a	C8 group		
HA86	2019-12-12	Heron	Pam's Point	*Stylophora*	*pistillata*	C8a	C8 group		
HA87	2019-12-12	Heron	Pam's	*Stylophora*	*pistillata*	C8a	C8 group		

			Point						
HA88	2019-12-12	Heron	Pam's Point	*Stylophora*	*pistillata*	C8a	C8 group		
HJ01	2019-12-04	Heron	Harry's Bommie	*Pocillopora*	*damicornis*	C33a*	C33a	*Symbiodinium*	*Symbiodinium sp.*
HJ02	2019-12-04	Heron	Harry's Bommie	*Pocillopora*	*damicornis*	C33a*	C33a	*Symbiodinium*	*Symbiodinium sp.*
HJ03	2019-12-04	Heron	Harry's Bommie	*Pocillopora*	*damicornis*	C33a	C33a	*Cladocopium*	*Cladocopium latusorum*
HJ04	2019-12-04	Heron	Harry's Bommie	*Pocillopora*	*damicornis*	C33a*	C33a		
HJ05	2019-12-04	Heron	Harry's Bommie	*Pocillopora*	*damicornis*	C33a*	C33a	*Symbiodinium*	*Symbiodinium sp.*
HJ06	2019-12-04	Heron	Harry's Bommie	*Pocillopora*	*damicornis*	C1b-c-42a	*C. latusorum*	*Cladocopium*	*Cladocopium latusorum*
HJ07	2019-12-04	Heron	Harry's Bommie	*Pocillopora*	*damicornis*	C33a*	C33a		
HJ08	2019-12-04	Heron	Harry's Bommie	*Pocillopora*	*damicornis*	C1b-c-42a	*C. latusorum*	*Cladocopium*	*Cladocopium latusorum*
HJ17	2019-12-08	Heron	Coral Canyons	*Pocillopora*	*damicornis*	C33a*	C33a	*Symbiodinium*	*Symbiodinium sp.*
HJ18	2019-12-08	Heron	Coral Canyons	*Pocillopora*	*damicornis*	C33a	C33a	*Cladocopium*	*Cladocopium latusorum*
HJ19	2019-12-08	Heron	Coral Canyons	*Pocillopora*	*damicornis*	C33a	C33a	*Symbiodinium*	*Symbiodinium sp.*

HJ20	2019-12-08	Heron	Coral Canyons	*Pocillopora*	*damicornis*	C33a	C33a	*Symbiodinium*	*Symbiodinium sp.*
HJ21	2019-12-08	Heron	Coral Canyons	*Pocillopora*	*damicornis*	C33a*	C33a	*Symbiodinium*	*Symbiodinium sp.*
HJ22	2019-12-08	Heron	Coral Canyons	*Pocillopora*	*damicornis*	C33a*	C33a	*Symbiodinium*	*Symbiodinium sp.*
HJ23	2019-12-08	Heron	Coral Canyons	*Pocillopora*	*damicornis*	C33a	C33a	*Symbiodinium*	*Symbiodinium sp.*
HJ24	2019-12-08	Heron	Coral Canyons	*Pocillopora*	*damicornis*	C33a*	C33a		
HJ33	2019-12-11	Heron	Pam's Point	*Pocillopora*	*damicornis*	C33a	C33a		
HJ34	2019-12-11	Heron	Pam's Point	*Pocillopora*	*damicornis*	C33a	C33a		
HJ35	2019-12-11	Heron	Pam's Point	*Pocillopora*	*damicornis*	C33a*	C33a	*Symbiodinium*	*Symbiodinium sp.*
HJ36	2019-12-11	Heron	Pam's Point	*Pocillopora*	*damicornis*	C33a*	C33a		
HJ37	2019-12-11	Heron	Pam's Point	*Pocillopora*	*damicornis*	C33a	C33a	*Symbiodinium*	*Symbiodinium sp.*
HJ38	2019-12-11	Heron	Pam's Point	*Pocillopora*	*damicornis*	C33a*	C33a	*Symbiodinium*	*Symbiodinium sp.*
HJ39	2019-12-11	Heron	Pam's Point	*Pocillopora*	*damicornis*	C33a*	C33a	*Symbiodinium*	*Symbiodinium sp.*
HJ40	2019-12-11	Heron	Pam's	*Pocillopora*	*damicornis*	C33a*	C33a	*Symbiodinium*	*Symbiodinium*

			Point						*sp.*
HJ49	2019-12-05	Heron	Harry's Bommie	*Stylophora*	*pistillata*	C78a	C78a		
HJ50	2019-12-05	Heron	Harry's Bommie	*Stylophora*	*pistillata*	C78a	C78a		
HJ51	2019-12-05	Heron	Harry's Bommie	*Stylophora*	*pistillata*	C78a	C78a		
HJ52	2019-12-05	Heron	Harry's Bommie	*Stylophora*	*pistillata*	C78a	C78a		
HJ53	2019-12-05	Heron	Harry's Bommie	*Stylophora*	*pistillata*	C78a	C78a		
HJ54	2019-12-05	Heron	Harry's Bommie	*Stylophora*	*pistillata*	C78a	C78a		
HJ55	2019-12-05	Heron	Harry's Bommie	*Stylophora*	*pistillata*	C78a	C78a		
HJ56	2019-12-05	Heron	Harry's Bommie	*Stylophora*	*pistillata*	C78a	C78a		
HJ65	2019-12-10	Heron	Coral Canyons	*Stylophora*	*pistillata*	C78a	C78a		
HJ66	2019-12-10	Heron	Coral Canyons	*Stylophora*	*pistillata*	C78a	C78a		
HJ67	2019-12-10	Heron	Coral Canyons	*Stylophora*	*pistillata*	C78a	C78a		
HJ68	2019-12-10	Heron	Coral Canyons	*Stylophora*	*pistillata*	C78a	C78a		

HJ69	2019-12-10	Heron	Coral Canyons	*Stylophora*	*pistillata*	C78a	C78a		
HJ70	2019-12-10	Heron	Coral Canyons	*Stylophora*	*pistillata*	C78a	C78a		
HJ71	2019-12-10	Heron	Coral Canyons	*Stylophora*	*pistillata*	C78a	C78a		
HJ72	2019-12-10	Heron	Coral Canyons	*Stylophora*	*pistillata*	C8a	C8 group		
HJ81	2019-12-12	Heron	Pam's Point	*Stylophora*	*pistillata*	C8a	C8 group		
HJ82	2019-12-12	Heron	Pam's Point	*Stylophora*	*pistillata*	C35a	C35a		
HJ83	2019-12-12	Heron	Pam's Point	*Stylophora*	*pistillata*	C35a?	C35a		
HJ84	2019-12-12	Heron	Pam's Point	*Stylophora*	*pistillata*	C35a	C35a		
HJ85	2019-12-12	Heron	Pam's Point	*Stylophora*	*pistillata*	C35a	C35a		
HJ86	2019-12-12	Heron	Pam's Point	*Stylophora*	*pistillata*	C35a+	C35a		
HJ87	2019-12-12	Heron	Pam's Point	*Stylophora*	*pistillata*	C35a	C35a		
HJ88	2019-12-12	Heron	Pam's Point	*Stylophora*	*pistillata*	C78a	C78a		
LA01	2019-11-18	Lizard	Granite	*Pocillopora*	*bairdi*	C1c-d-t	*C.*	*Cladocopium*	*Cladocopium*

			Bluff				*pacificum*		*pacificum*
LA02	2019-11-18	Lizard	Granite Bluff	*Pocillopora*	*verrucosa*	C1c-d-t	*C. pacificum*	*Cladocopium*	*Cladocopium pacificum*
LA03	2019-11-18	Lizard	Granite Bluff	*Pocillopora*	*verrucosa*	C1c-d-t	*C. pacificum*	*Cladocopium*	*Cladocopium pacificum*
LA04	2019-11-18	Lizard	Granite Bluff	*Pocillopora*	*bairdi*	C1c-d-t	*C. pacificum*	*Cladocopium*	*Cladocopium pacificum*
LA05	2019-11-18	Lizard	Granite Bluff	*Pocillopora*	*bairdi*	C1c-d-t	*C. pacificum*		
LA06	2019-11-18	Lizard	Granite Bluff	*Pocillopora*	*verrucosa*	C1c-d-t	*C. pacificum*	*Cladocopium*	*Cladocopium pacificum*
LA07	2019-11-18	Lizard	Granite Bluff	*Pocillopora*	*bairdi*	C1c-d-t	*C. pacificum*	*Cladocopium*	*Cladocopium pacificum*
LA08	2019-11-18	Lizard	Granite Bluff	*Pocillopora*	*verrucosa*	C1c-d-t	*C. pacificum*	*Cladocopium*	*Cladocopium pacificum*
LA13	2019-11-20	Lizard	Big Vicki's	*Pocillopora*	*bairdi*	C1c-d-t	*C. pacificum*		
LA14	2019-11-20	Lizard	Big Vicki's	*Pocillopora*	*bairdi*	C1c-d-t	*C. pacificum*	*Cladocopium*	*Cladocopium pacificum*
LA15	2019-11-20	Lizard	Big Vicki's	*Pocillopora*	*verrucosa*	C1c-d-t	*C. pacificum*	*Cladocopium*	*Cladocopium pacificum*
LA16	2019-11-20	Lizard	Big Vicki's	*Pocillopora*	*verrucosa*	C1c-d-t	*C. pacificum*	*Cladocopium*	*Cladocopium pacificum*
LA17	2019-11-20	Lizard	Big Vicki's	*Pocillopora*	*bairdi*	C1c-d	*C. pacificum*	*Cladocopium*	*Cladocopium pacificum*

LA18	2019-11-20	Lizard	Big Vicki's	*Pocillopora*	*bairdi*	C1c-d-t	*C. pacificum*	*Cladocopium*	*Cladocopium pacificum*
LA19	2019-11-20	Lizard	Big Vicki's	*Pocillopora*	*bairdi*	C1c-d-t	*C. pacificum*	*Cladocopium*	*Cladocopium pacificum*
LA20	2019-11-20	Lizard	Big Vicki's	*Pocillopora*	*verrucosa*	C1c-d-t	*C. pacificum*	*Cladocopium*	*Cladocopium pacificum*
LA25	2019-11-23	Lizard	Turtle South	*Pocillopora*	*verrucosa*	C1c-d	*C. pacificum*	*Cladocopium*	*Cladocopium pacificum*
LA26	2019-11-23	Lizard	Turtle South	*Pocillopora*	*bairdi*	C1c-d	*C. pacificum*		
LA27	2019-11-23	Lizard	Turtle South	*Pocillopora*	*bairdi*	C1c-d	*C. pacificum*	*Cladocopium*	*Cladocopium pacificum*
LA28	2019-11-23	Lizard	Turtle South	*Pocillopora*	*bairdi*	C1c-d	*C. pacificum*		
LA29	2019-11-23	Lizard	Turtle South	*Pocillopora*	*bairdi*	C1c-d	*C. pacificum*	*Cladocopium*	*Cladocopium pacificum*
LA30	2019-11-23	Lizard	Turtle South	*Pocillopora*	*verrucosa*	C1c-d	*C. pacificum*		
LA31	2019-11-23	Lizard	Turtle South	*Pocillopora*	*verrucosa*	C1c-d	*C. pacificum*	*Cladocopium*	*Cladocopium pacificum*
LA32	2019-11-23	Lizard	Turtle South	*Pocillopora*	*verrucosa*	C1c-d	*C. pacificum*	*Cladocopium*	*Cladocopium pacificum*
LA49	2019-11-19	Lizard	Granite Bluff	*Stylophora*	*pistillata*	C8b	C8 group		
LA50	2019-11-19	Lizard	Granite	*Stylophora*	*pistillata*	C8b	C8 group		

			Bluff						
LA51	2019-11-19	Lizard	Granite Bluff	*Stylophora*	*pistillata*	C8b	C8 group		
LA52	2019-11-19	Lizard	Granite Bluff	*Stylophora*	*pistillata*	C8b	C8 group		
LA53	2019-11-19	Lizard	Granite Bluff	*Stylophora*	*pistillata*	C8-unk	C8 group		
LA54	2019-11-19	Lizard	Granite Bluff	*Stylophora*	*pistillata*	C8b	C8 group		
LA55	2019-11-19	Lizard	Granite Bluff	*Stylophora*	*pistillata*	C8b	C8 group		
LA56	2019-11-19	Lizard	Granite Bluff	*Stylophora*	*pistillata*	C8b	C8 group		
LA65	2019-11-21	Lizard	Big Vicki's	*Stylophora*	*pistillata*	C8b	C8 group		
LA66	2019-11-21	Lizard	Big Vicki's	*Stylophora*	*pistillata*	C8-unk	C8 group		
LA67	2019-11-21	Lizard	Big Vicki's	*Stylophora*	*pistillata*	C8b	C8 group		
LA68	2019-11-21	Lizard	Big Vicki's	*Stylophora*	*pistillata*	C8-unk	C8 group		
LA69	2019-11-21	Lizard	Big Vicki's	*Stylophora*	*pistillata*	C8-unk	C8 group		
LA70	2019-11-21	Lizard	Big Vicki's	*Stylophora*	*pistillata*	C8b	C8 group		

LA71	2019-11-22	Lizard	Big Vicki's	*Stylophora*	*pistillata*	C8-unk	C8 group		
LA72	2019-11-22	Lizard	Big Vicki's	*Stylophora*	*pistillata*	C8-unk	C8 group		
LA81	2019-11-24	Lizard	Turtle South	*Stylophora*	*pistillata*	C8b	C8 group		
LA82	2019-11-24	Lizard	Turtle South	*Stylophora*	*pistillata*	C8b	C8 group		
LA83	2019-11-24	Lizard	Turtle South	*Stylophora*	*pistillata*	C8b	C8 group		
LA84	2019-11-24	Lizard	Turtle South	*Stylophora*	*pistillata*	C8b	C8 group		
LA85	2019-11-24	Lizard	Turtle South	*Stylophora*	*pistillata*	C8b	C8 group		
LA86	2019-11-24	Lizard	Turtle South	*Stylophora*	*pistillata*	C8b	C8 group		
LA87	2019-11-24	Lizard	Turtle South	*Stylophora*	*pistillata*	C8b	C8 group		
LA88	2019-11-24	Lizard	Turtle South	*Stylophora*	*pistillata*	C8b	C8 group		
LJ01	2019-11-18	Lizard	Granite Bluff	*Pocillopora*	*verrucosa*	C1c	*C. pacificum*	*Cladocopium*	*Cladocopium pacificum*
LJ02	2019-11-18	Lizard	Granite Bluff	*Pocillopora*	*bairdi*	C1c	*C. pacificum*	*Cladocopium*	*Cladocopium pacificum*
LJ03	2019-11-18	Lizard	Granite	*Pocillopora*	*verrucosa*	C1c-d-t	*C.*	*Cladocopium*	*Cladocopium*

			Bluff				*pacificum*		*pacificum*
LJ04	2019-11-18	Lizard	Granite Bluff	*Pocillopora*	*bairdi*	C1c-d-t	*C. pacificum*	*Cladocopium*	*Cladocopium pacificum*
LJ05	2019-11-18	Lizard	Granite Bluff	*Pocillopora*	*bairdi*	C1c-d-t	*C. pacificum*	*Cladocopium*	*Cladocopium pacificum*
LJ06	2019-11-18	Lizard	Granite Bluff	*Pocillopora*	*bairdi*	C1c-d-t, D1a	*C. pacificum*	*Cladocopium*	*Cladocopium pacificum*
LJ07	2019-11-18	Lizard	Granite Bluff	*Pocillopora*	*bairdi*	C1c-d-t	*C. pacificum*		
LJ08	2019-11-18	Lizard	Granite Bluff	*Pocillopora*	*bairdi*	C1c-d-t	*C. pacificum*	*Cladocopium*	*Cladocopium pacificum*
LJ13	2019-11-20	Lizard	Big Vicki's	*Pocillopora*	*verrucosa*	C1c-d-t	*C. pacificum*	*Cladocopium*	*Cladocopium pacificum*
LJ14	2019-11-20	Lizard	Big Vicki's	*Pocillopora*	*verrucosa*	C1c-d-t	*C. pacificum*	*Cladocopium*	*Cladocopium pacificum*
LJ15	2019-11-20	Lizard	Big Vicki's	*Pocillopora*	*bairdi*	C1c-d-t	*C. pacificum*	*Cladocopium*	*Cladocopium pacificum*
LJ16	2019-11-20	Lizard	Big Vicki's	*Pocillopora*	*bairdi*	C1c-d-t	*C. pacificum*		
LJ17	2019-11-20	Lizard	Big Vicki's	*Pocillopora*	*acuta*	C1c-d-t	*C. pacificum*	*Symbiodinium*	*Symbiodinium sp.*
LJ18	2019-11-20	Lizard	Big Vicki's	*Pocillopora*	*bairdi*	C1c-d-t	*C. pacificum*	*Cladocopium*	*Cladocopium pacificum*
LJ19	2019-11-20	Lizard	Big Vicki's	*Pocillopora*	*verrucosa*	C1c-d-t	*C. pacificum*	*Cladocopium*	*Cladocopium pacificum*

LJ20	2019-11-20	Lizard	Big Vicki's	*Pocillopora*	*acuta*	C1cc	*C. pacificum*	*Cladocopium*	*Cladocopium pacificum*
LJ25	2019-11-23	Lizard	Turtle South	*Pocillopora*	*acuta*	C1c-d	*C. pacificum*	*Cladocopium*	*Cladocopium pacificum*
LJ26	2019-11-23	Lizard	Turtle South	*Pocillopora*	*verrucosa*	C1c-d-t	*C. pacificum*	*Cladocopium*	*Cladocopium pacificum*
LJ27	2019-11-23	Lizard	Turtle South	*Pocillopora*	*bairdi*	C1c-d-t	*C. pacificum*	*Cladocopium*	*Cladocopium pacificum*
LJ28	2019-11-23	Lizard	Turtle South	*Pocillopora*	*acuta*	C1c-d-t	*C. pacificum*	*Cladocopium*	*Cladocopium pacificum*
LJ29	2019-11-23	Lizard	Turtle South	*Pocillopora*	*bairdi*	C1c-d-t	*C. pacificum*	*Cladocopium*	*Cladocopium pacificum*
LJ30	2019-11-23	Lizard	Turtle South	*Pocillopora*	*acuta*	C1cc	*C. pacificum*		
LJ31	2019-11-23	Lizard	Turtle South	*Pocillopora*	*verrucosa*	C1c-d-t	*C. pacificum*	*Cladocopium*	*Cladocopium pacificum*
LJ32	2019-11-23	Lizard	Turtle South	*Pocillopora*	*verrucosa*	C1c-d-t	*C. pacificum*	*Cladocopium*	*Cladocopium pacificum*
LJ49	2019-11-19	Lizard	Granite Bluff	*Stylophora*	*pistillata*	C8	C8 group		
LJ50	2019-11-19	Lizard	Granite Bluff	*Stylophora*	*pistillata*	C8	C8 group		
LJ51	2019-11-19	Lizard	Granite Bluff	*Stylophora*	*pistillata*	C8	C8 group		
LJ52	2019-11-19	Lizard	Granite	*Stylophora*	*pistillata*	C8	C8 group		

			Bluff						
LJ53	2019-11-19	Lizard	Granite Bluff	*Stylophora*	*pistillata*	C8	C8 group		
LJ54	2019-11-19	Lizard	Granite Bluff	*Stylophora*	*pistillata*	C8b	C8 group		
LJ55	2019-11-19	Lizard	Granite Bluff	*Stylophora*	*pistillata*	C8	C8 group		
LJ56	2019-11-19	Lizard	Granite Bluff	*Stylophora*	*pistillata*	C8b	C8 group		
LJ65	2019-11-21	Lizard	Big Vicki's	*Stylophora*	*pistillata*	C8b	C8 group		
LJ66	2019-11-21	Lizard	Big Vicki's	*Stylophora*	*pistillata*	C8-unk	C8 group		
LJ67	2019-11-21	Lizard	Big Vicki's	*Stylophora*	*pistillata*	C8-unk	C8 group		
LJ68	2019-11-21	Lizard	Big Vicki's	*Stylophora*	*pistillata*	C8	C8 group		
LJ69	2019-11-21	Lizard	Big Vicki's	*Stylophora*	*pistillata*	C8-unk	C8 group		
LJ70	2019-11-21	Lizard	Big Vicki's	*Stylophora*	*pistillata*	C8-unk	C8 group		
LJ71	2019-11-22	Lizard	Big Vicki's	*Stylophora*	*pistillata*	C8-unk	C8 group		
LJ72	2019-11-22	Lizard	Big Vicki's	*Stylophora*	*pistillata*	C8-unk	C8 group		

LJ81	2019-11-24	Lizard	Turtle South	*Stylophora*	*pistillata*	C8b	C8 group		
LJ82	2019-11-24	Lizard	Turtle South	*Stylophora*	*pistillata*	C8b	C8 group		
LJ83	2019-11-24	Lizard	Turtle South	*Stylophora*	*pistillata*	C8	C8 group		
LJ84	2019-11-24	Lizard	Turtle South	*Stylophora*	*pistillata*	C8	C8 group		
LJ85	2019-11-24	Lizard	Turtle South	*Stylophora*	*pistillata*	C8	C8 group		
LJ86	2019-11-24	Lizard	Turtle South	*Stylophora*	*pistillata*	C8b	C8 group		
LJ87	2019-11-24	Lizard	Turtle South	*Stylophora*	*pistillata*	C8b	C8 group		
LJ88	2019-11-24	Lizard	Turtle South	*Stylophora*	*pistillata*	C8	C8 group		

Table S5. Summary of $\delta^{13}C_{host\text{-}symbiont}$ ($\Delta^{13}C$) and $\delta^{15}N_{host\text{-}symbiont}$ ($\Delta^{15}N$) values for corals at the holobiont level (mean ± SD) before (November–December 2019) the marine heatwave and mass coral bleaching of 2020.

Location	Coral holobiont	Colony size	n	$\Delta^{13}C$ (‰)	$\Delta^{15}N$ (‰)
Lizard Island	*S. pistillata*, C8 group	Juvenile	12	-0.38 ± 0.42	0.85 ± 0.55
		Adult	12	-0.63 ± 0.43	0.65 ± 0.52
	P. verrucosa, *C. pacificum*	Adult	3	-1.00 ± 0.40	0.80 ± 0.35
		Juvenile	4	-0.35 ± 0.26	1.98 ± 1.21
	P. bairdi, *C. pacificum*	Adult	5	-0.46 ± 0.11	1.40 ± 0.46
		Juvenile	4	-0.18 ± 0.22	0.53 ± 0.17
	P. acuta, *C. pacificum*	Juvenile	3	-0.50 ± 0.82	0.63 ± 1.00
Heron Island	*P. damicornis*, C33a_sGBR	Adult	11	-0.03 ± 1.99	1.33 ± 0.67
		Juvenile	8	0.55 ± 0.65	1.79 ± 0.96
	S. pistillata, C78a	Juvenile	4	0.85 ± 1.11	0.70 ± 0.53
		Adult	9	0.08 ± 0.45	0.94 ± 0.69
	S. pistillata, C35a	Juvenile	3	-0.10 ± 0.44	0.53 ± 0.25
	S. pistillata, C8 groupH	Adult	5	-0.14 ± 0.15	1.34 ± 0.59

Supplemental figures

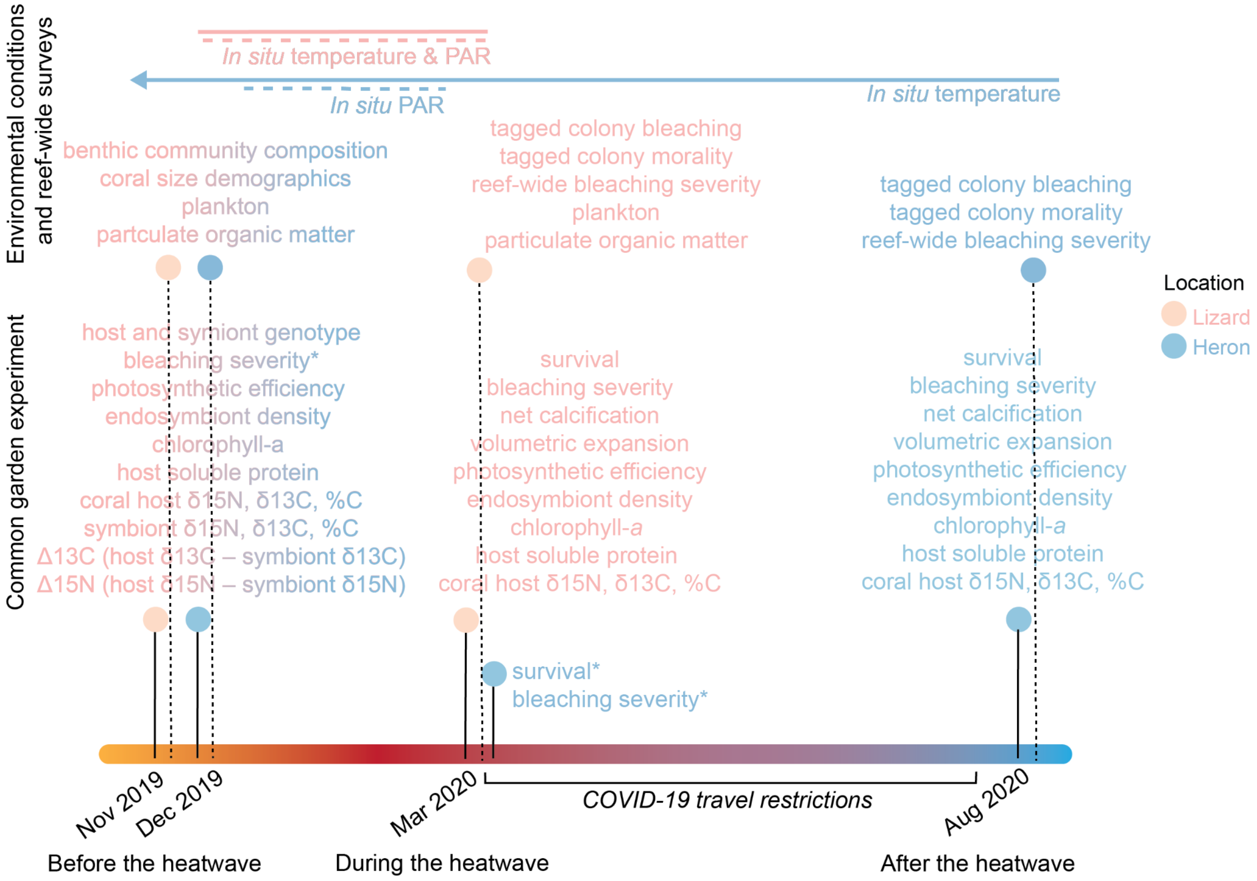

Figure S1. Detailed experimental timeline indicating when measurements were taken at each location. Asterisk (*) indicate parameters determined through photographs.

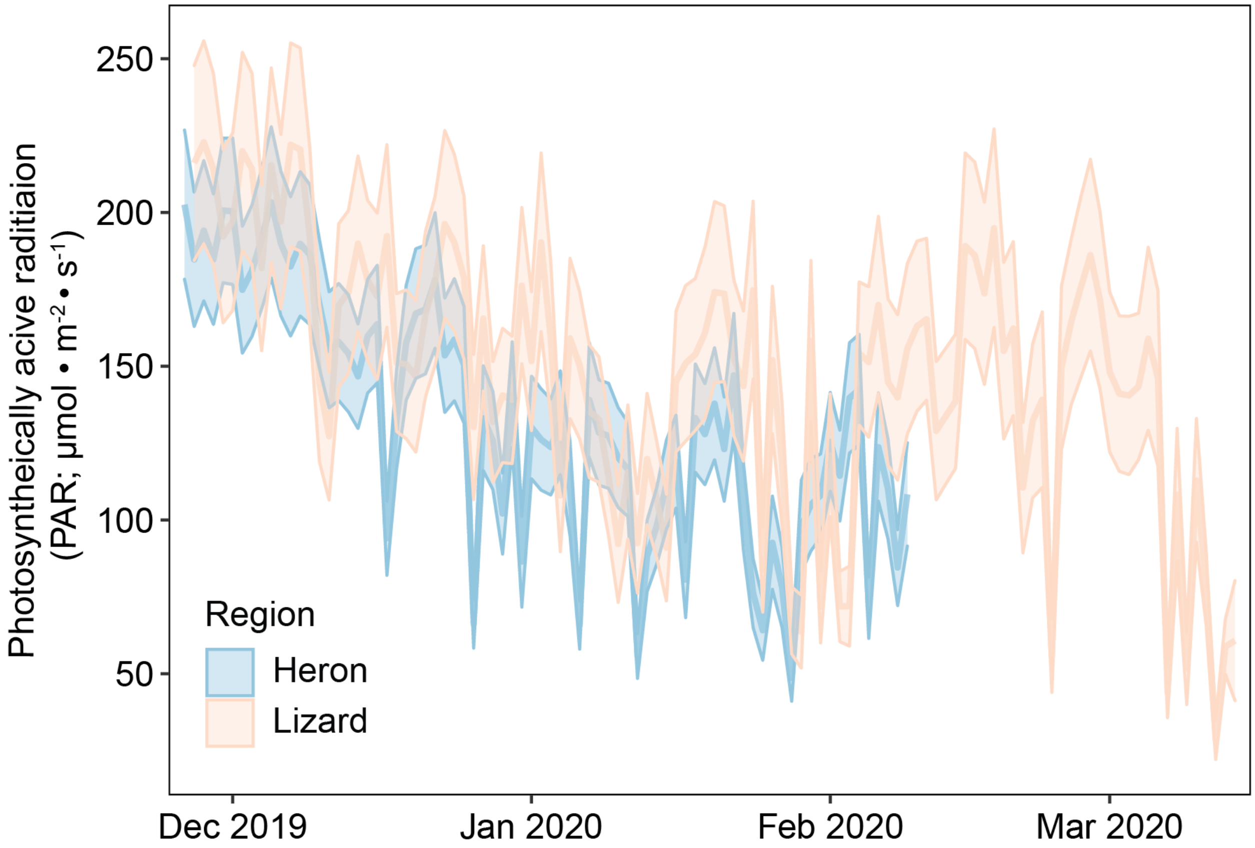

Figure S2. *In situ* photosynthetically active radiation (mean ± SE; n=3 sites per region) recorded at a depth of 5–8 m across the Great Barrier Reef between November 2019 and March 2020.

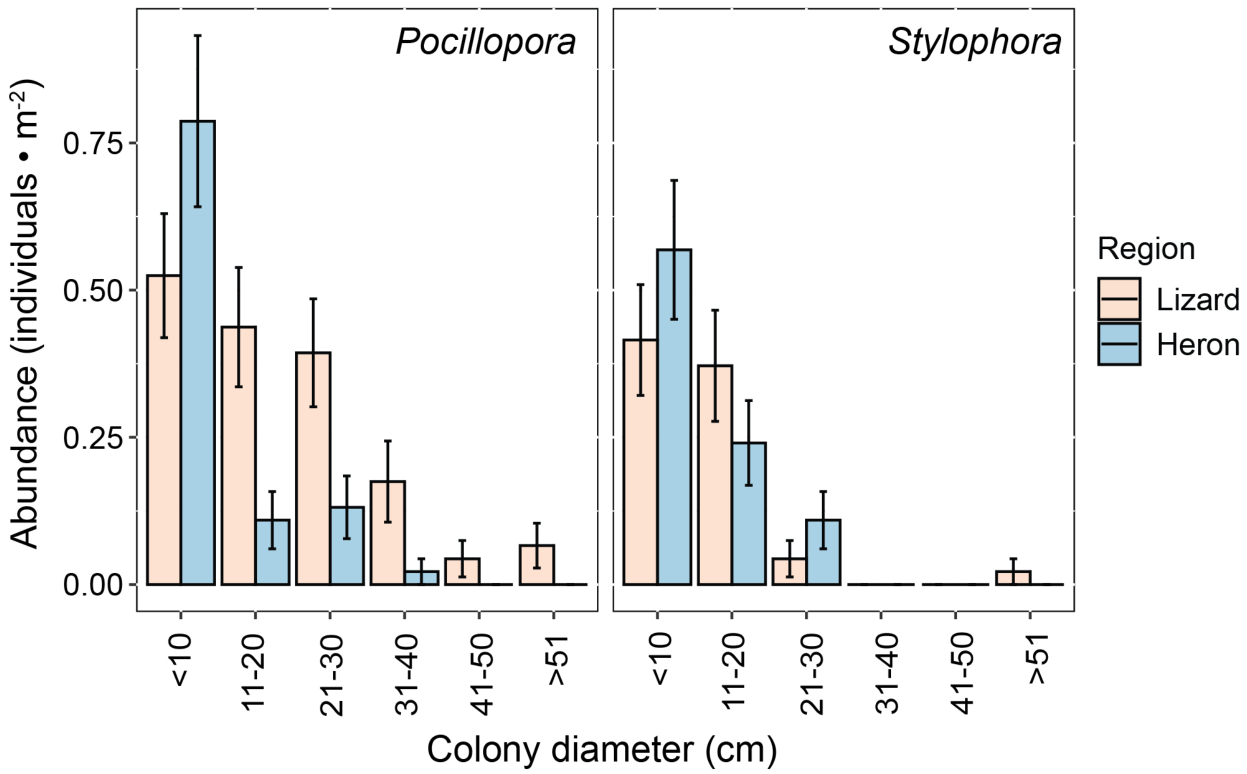

Figure S3. The size demographics of *Pocillopora* and *Stylophora* between regions.

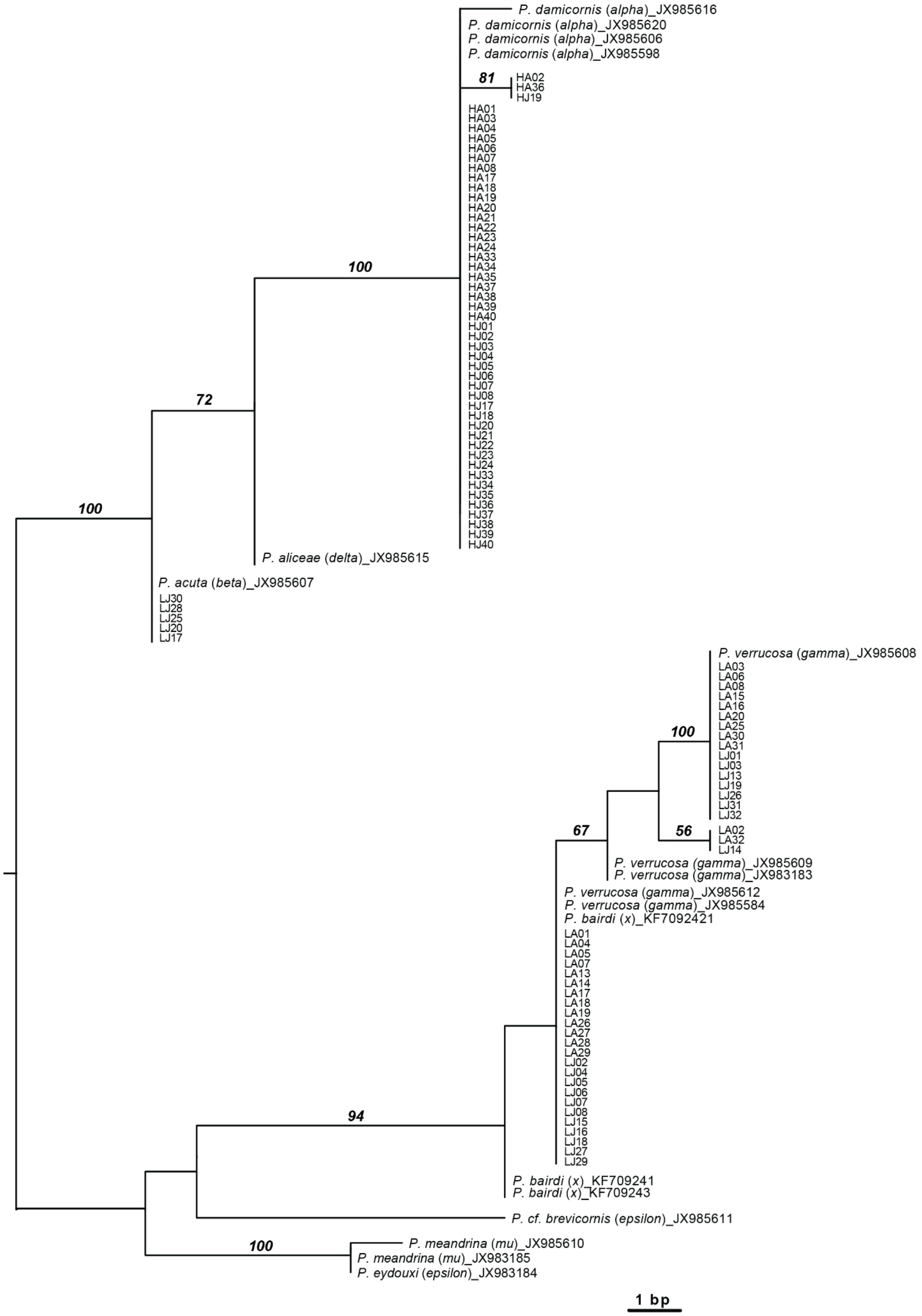

Figure S4. Maximum Parsimony tree constructed from *Pocillopora* mitochondrial ORF sequences (Flot et al., 2008). References are included for confirmed *Pocillopora* species with their GenBank accession numbers (Schmidt-Roach et al., 2014). Bootstrap values over 50 are shown by respective branches and branch lengths represent the number of base changes between sequences.

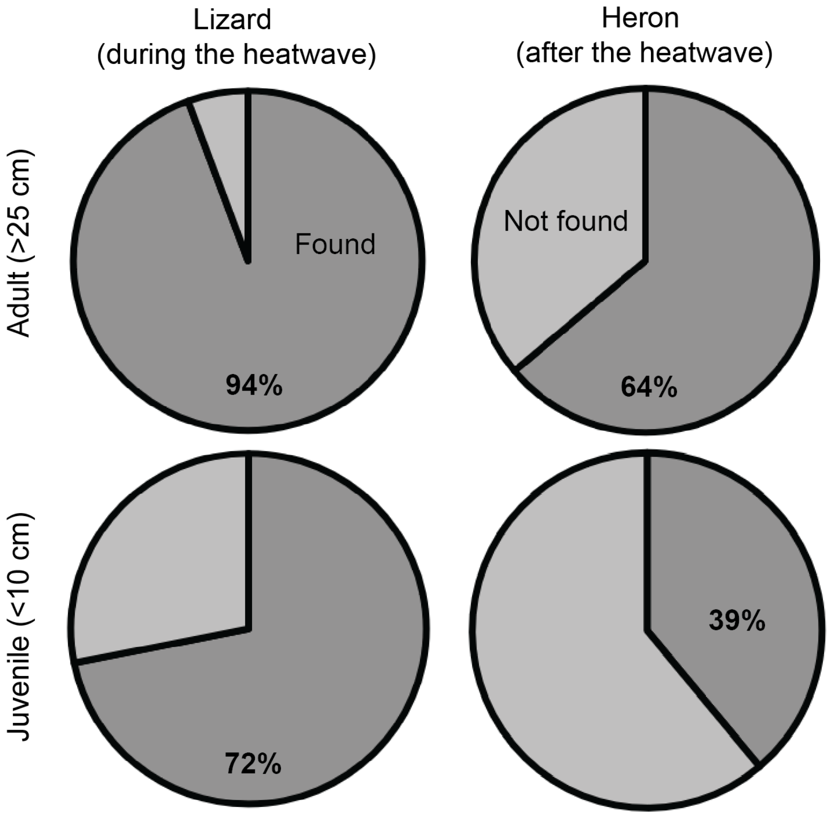

Figure S5. Proportion of tagged *Pocillopora* colonies found across the Great Barrier Reef during (Lizard Island) and after (Heron Island) the 2020 marine heatwave. Percentage (%) indicates the colonies found (dark gray), where the proportion of colonies not found are in light gray.

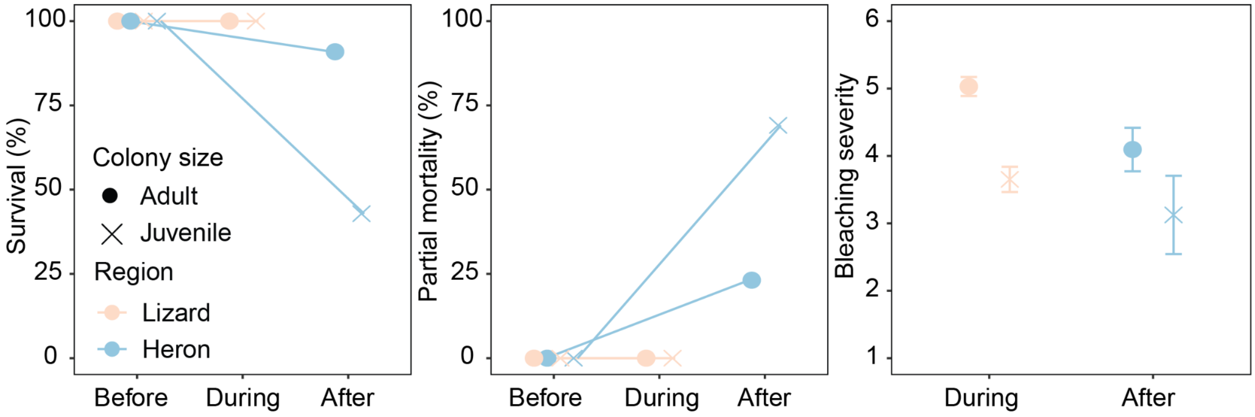

Figure S6. Survival, partial morality and bleaching severity of tagged *Pocillopora* colonies found across the Great Barrier Reef during (Lizard Island) and after (Heron Island) the 2020 marine heatwave.

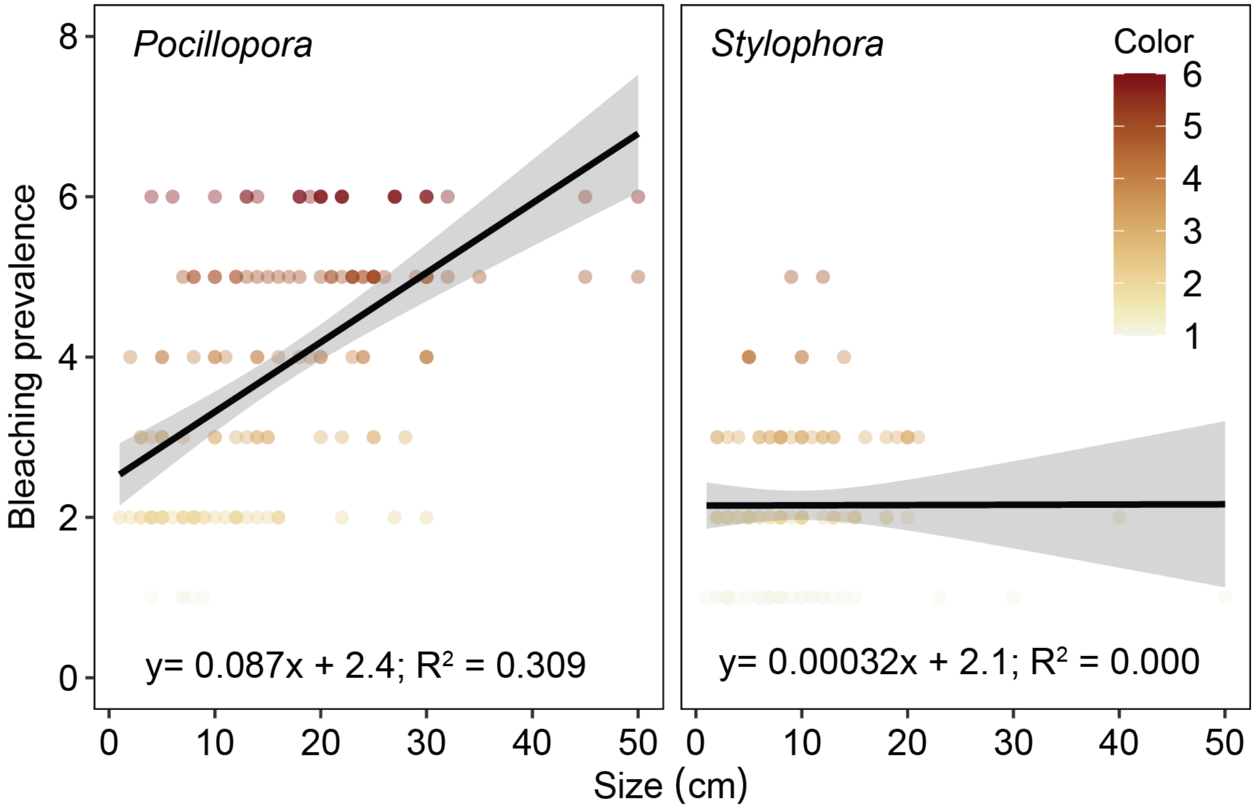

Figure S7. Relationship (± 95% CI) between reef-wide bleaching prevalence and colony size for *Pocillopora* and *Stylophora* during the 2020 heatwave (March 2020) at Lizard Island.

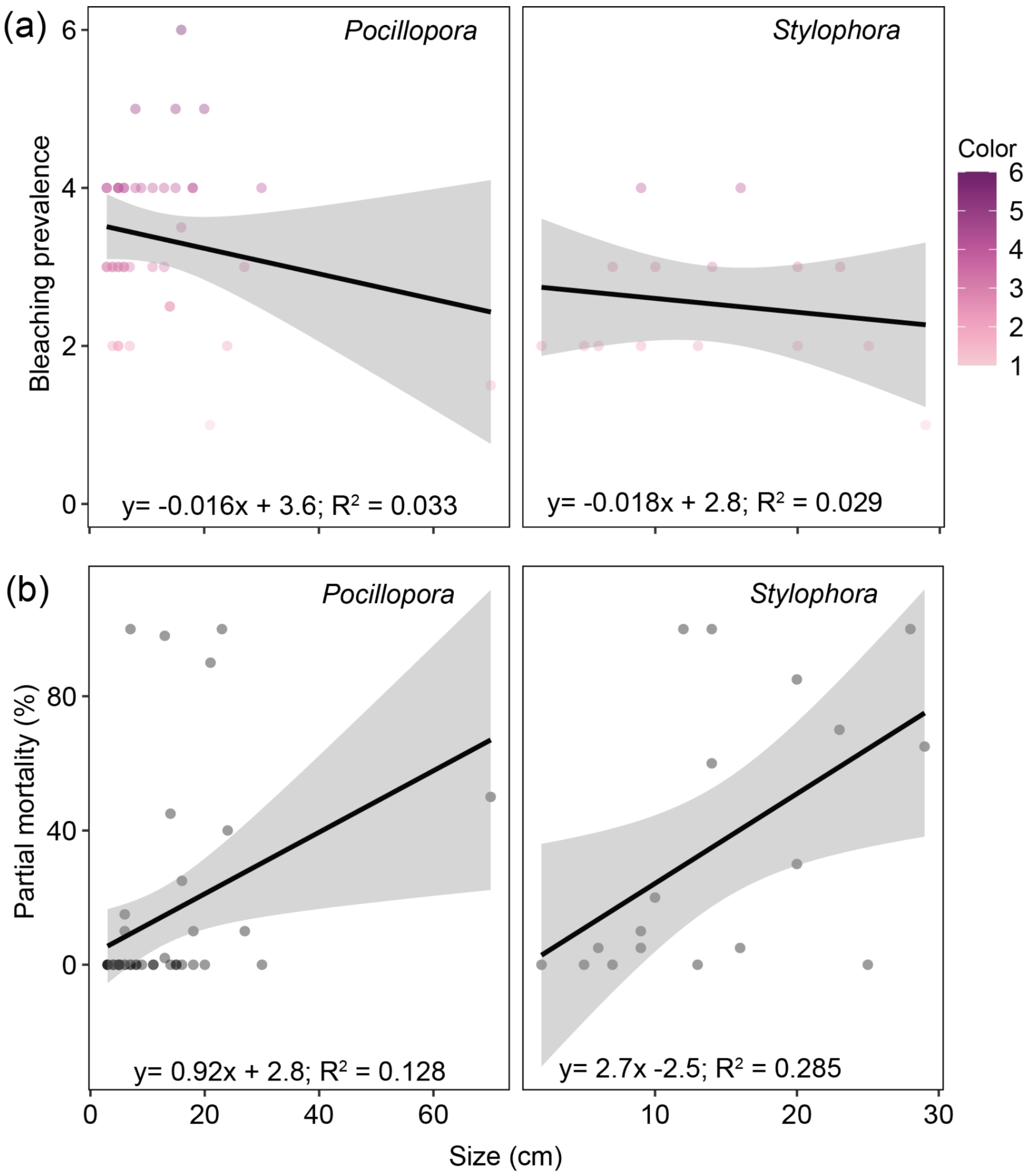

Figure S8. Relationship (± 95% CI) between reef-wide (a) bleaching prevalence and colony size and (b) partial mortality and colony size for *Pocillopora* and *Stylophora* after the 2020 heatwave (August 2020) at Heron Island.

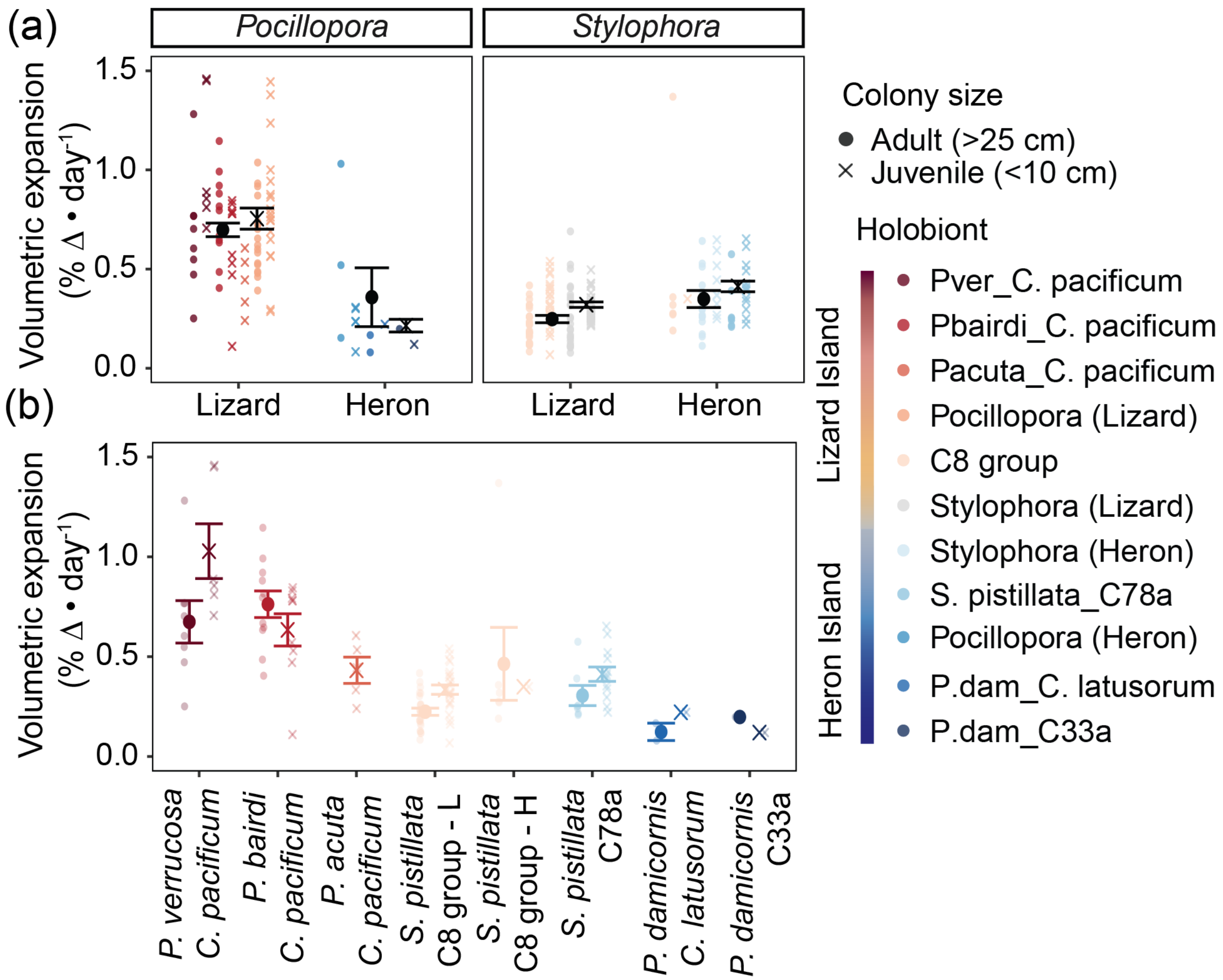

Figure S9. Volumetric expansion across the Great Barrier Reef by region, colony size and holobiont. (a) Relative change in volumetric expansion (mean ± SE) by region, coral genus and colony size. (b) Relative change in volumetric expansion (mean ± SE) by colony size and holobiont. Points represent individual coral fragments.

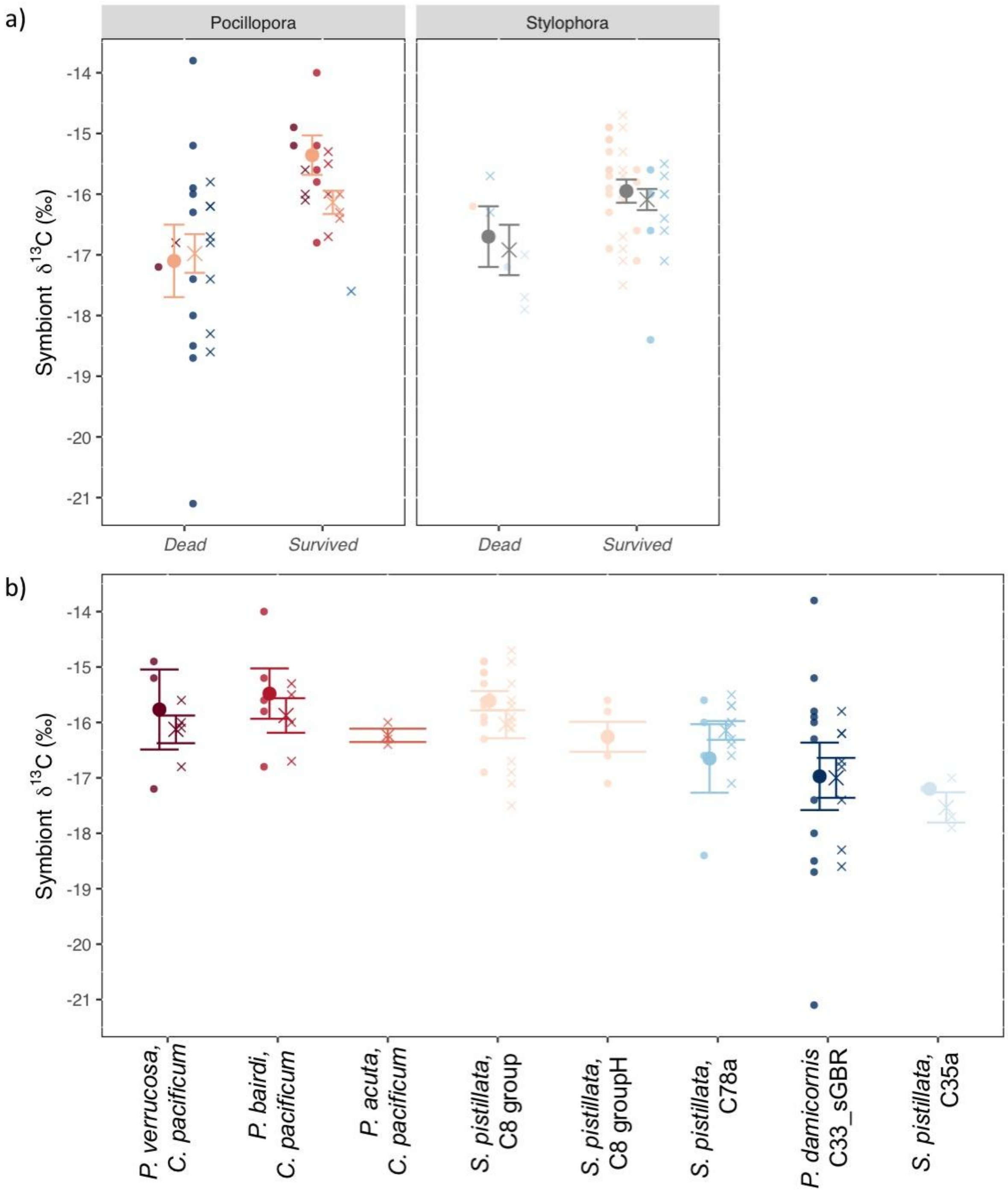

Figure S10. Symbiont carbon isotope ($\delta^{13}C$) values from before the marine heatwave on the Great Barrier Reef presented by a) coral genus and the 2020 heatwave survival outcome (died or survived) of individual coral fragment from adult (circle) and juvenile (x) colonies. b) Symbiont $\delta^{13}C$ values by holobiont genotype from Lizard Island (warm colors, four left holobiont genotypes) and Heron Island (four right holobiont genotypes) before the 2020 marine heatwave. Same holobiont color palette as presented in Figure 4.

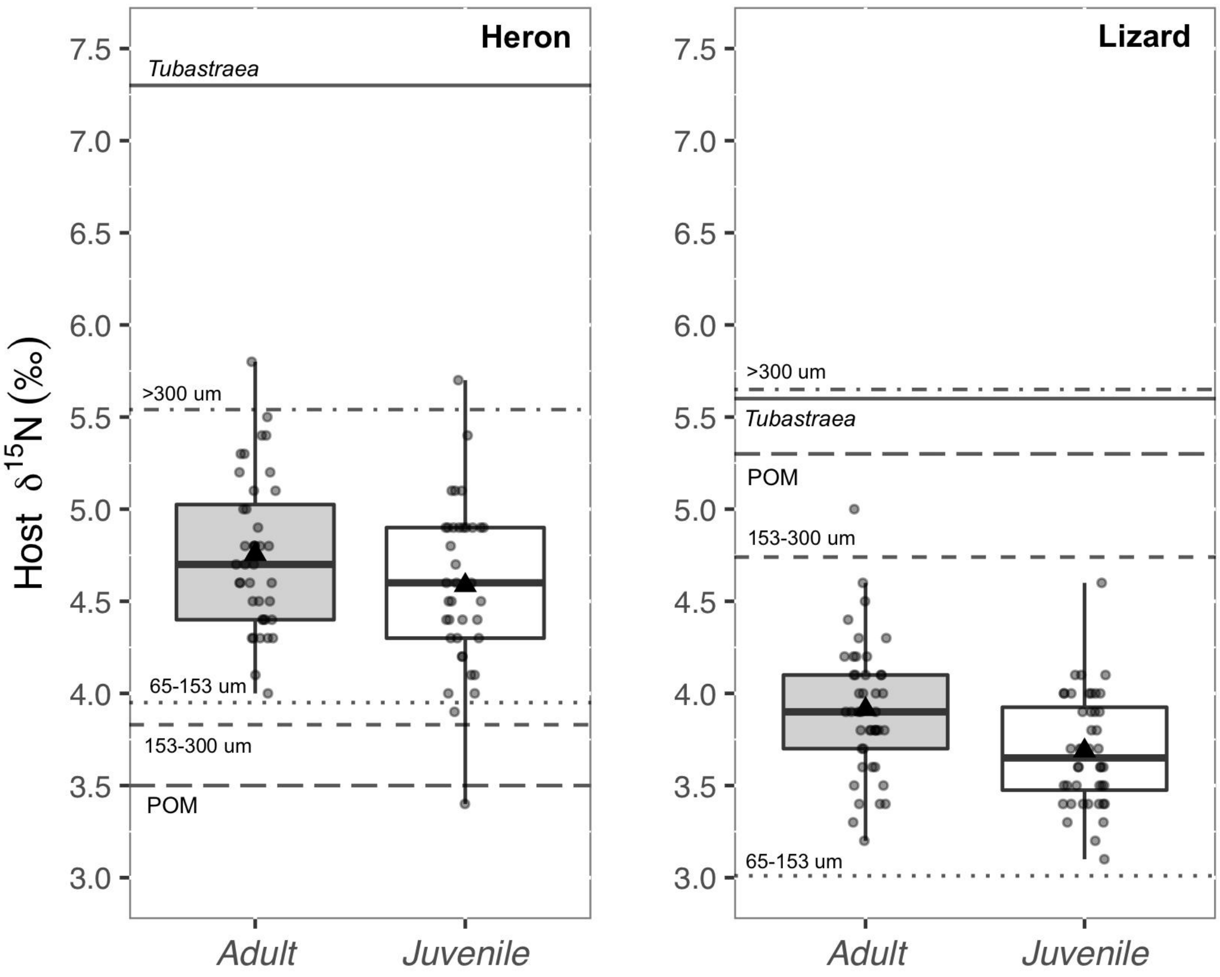

Figure S11. Coral host nitrogen stable isotope values ($\delta^{15}N$) of adult and juvenile corals from Heron Island (southern Great Barrier Reef; December 2019) and Lizard Island (northern GBR; November 2019) with environmental resource $\delta^{15}N$ data including three plankton size fractions (horizontal dashed lines), POM (largest dashed horizontal line), and non-symbiotic, heterotrophic coral *Tubastraea* cf. *coccinea* (solid horizontal line). Black triangles depict the mean values.

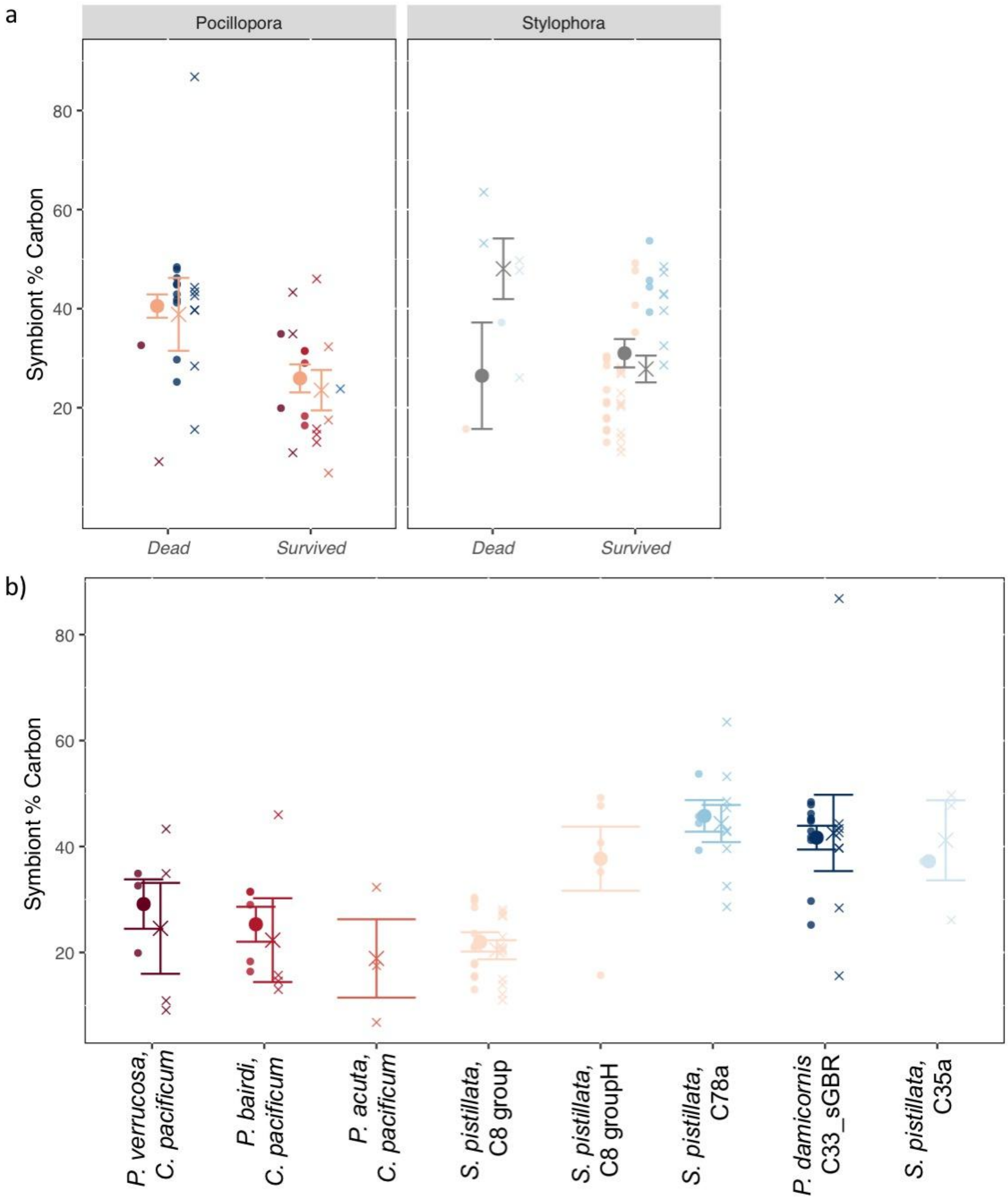

Figure S12. Symbiont tissue carbon content (%C) from adult (circle) and juvenile (x) corals on the Great Barrier Reef in November–December 2019 (before the 2020 marine heatwave). a) Before bleaching symbiont %C in *Pocillopora* and *Stylophora* presented by the ultimate survival outcome of the 2020 mass coral bleaching. b) Overall lower symbiont %C in individual coral fragments from Lizard Island (four left holobiont genotypes, warm colors) and Heron Island (four right holobiont genotypes) before the 2020 marine heatwave. Same holobiont color palette as presented in Figure 4.

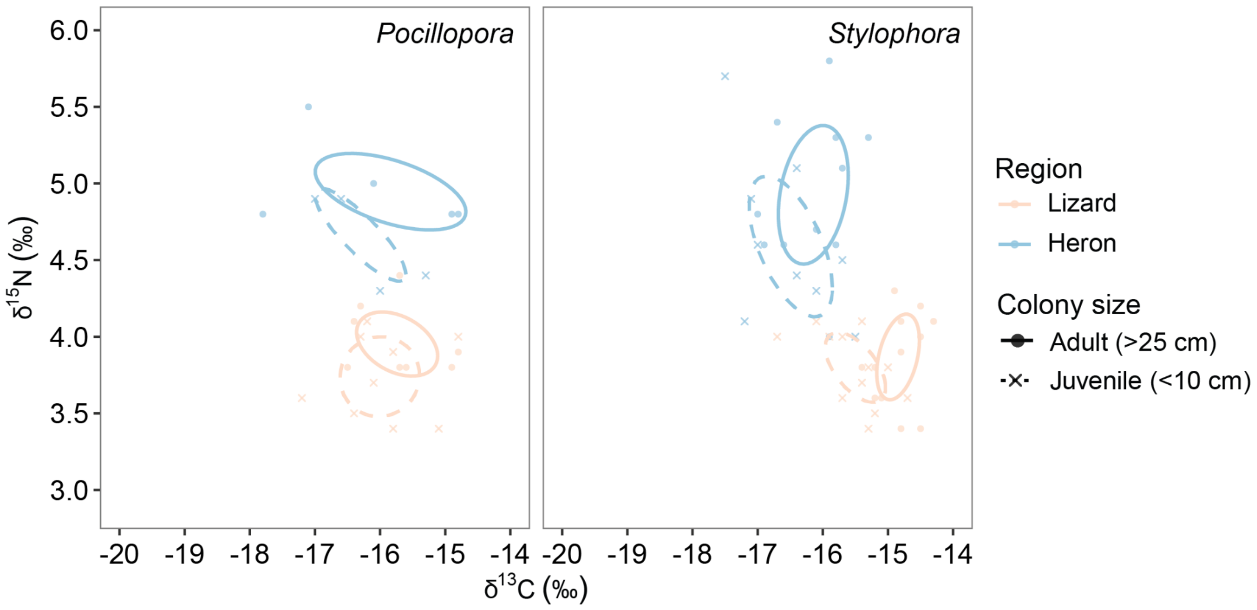

Figure S13. Coral host tissue stable isotopes from during/after the 2020 marine heatwave on the Great Barrier Reef. Isotopic niches of coral hosts *Pocillopora* and *Stylophora* of adult and juvenile size classes from Lizard Island (during) and Heron Island (after). Isotopic niches represent the standard ellipse areas comprising core data (40%).

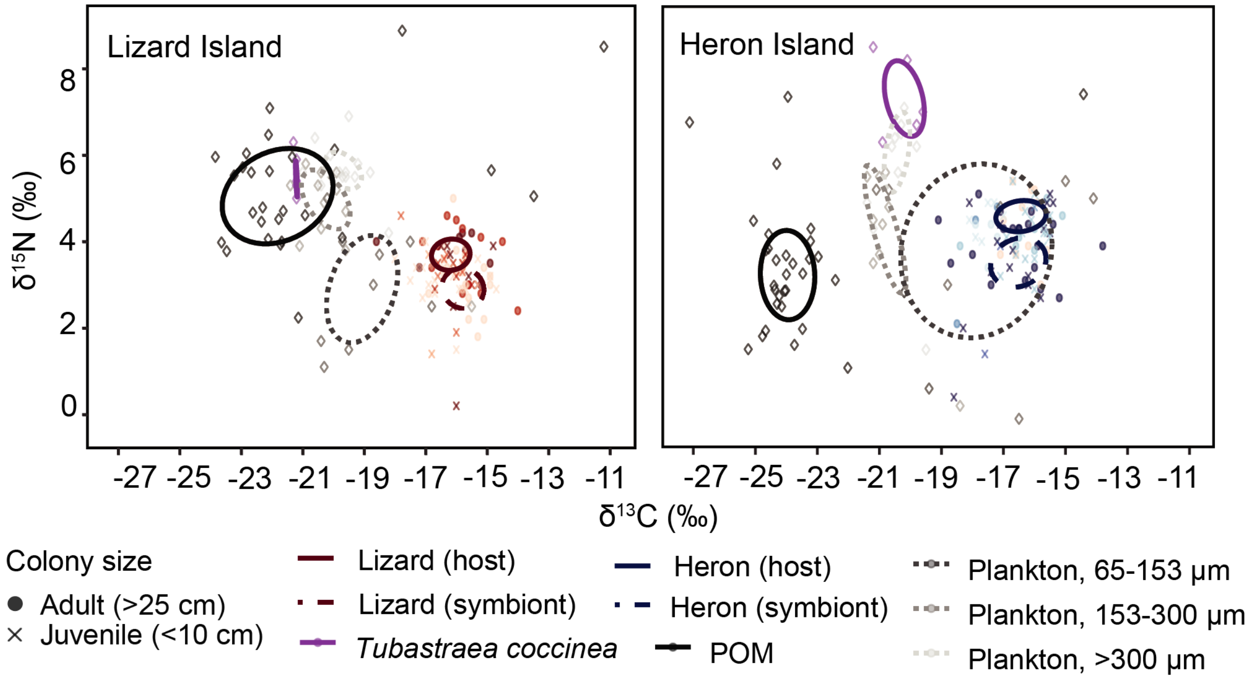

Figure S14. Isotopic niches of coral hosts, symbionts, and particulate resources (particulate organic matter=POM <0.7 µm; plankton, three size fractions) at Lizard Island (left) and Heron Island (right) on the Great Barrier Reef before (November–December 2019) the marine heatwave in early 2020. The non-symbiotic, scleractinian coral (*Tubastraea* cf. *coccinea*) served as a local heterotrophic baseline for each location. Isotopic niches represent the standard ellipse areas comprising core data (40%).

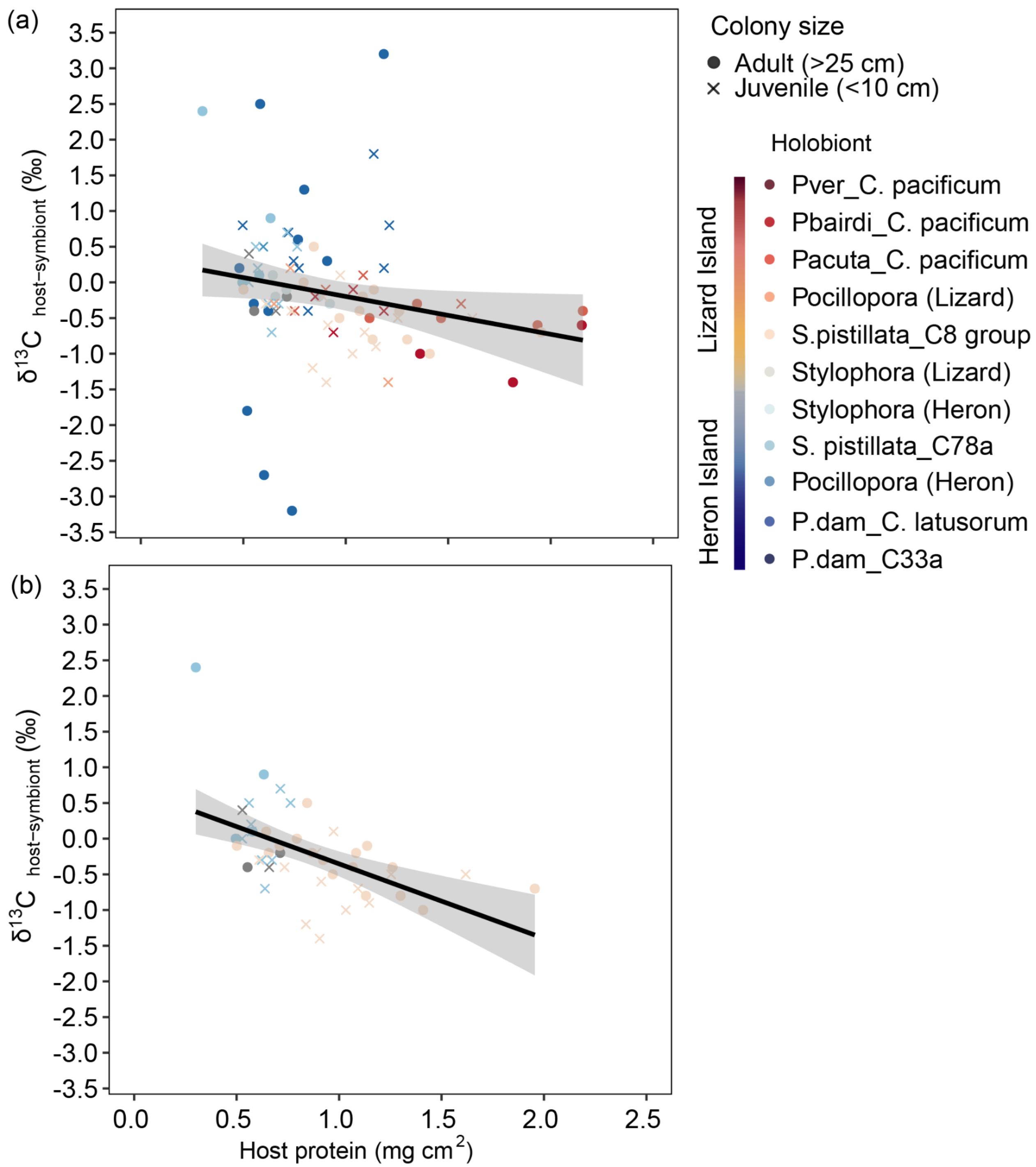

Figure S15. Relationship (± 95% CI) between coral host protein concentrations and the difference in carbon stable isotopes between coral hosts and symbionts ($\delta^{13}C_{host\text{-}symbiont}$, or commonly $\Delta^{13}C$) in November–December 2019 (before the 2020 marine heatwave) for a) all holobiont combinations at Heron Island and Lizard Island and b) *Stylophora pistillata–Cladocopium* 'C8 group' holobiont (the only host-Symbiodiniaceae combination

present in both locations). Points indicate individual samples, where Heron- blue, Lizard- peach, no symbiont ID- grey .

References

Bates, D., Maechler, M., Bolker, B., Walker, S., Christensen, R. H. B., Singmann, H., Dai, B., Grothendieck, G., Eigen, C., & Rcpp, L. (2015). Package "lme4." *Convergence*, *12*(1).

Bryant, D. E. P., Rodriguez-Ramirez, A., Phinn, S., González-Rivero, M., Brown, K. T., Neal, B. P., Hoegh-Guldberg, O., & Dove, S. (2017). Comparison of two photographic methodologies for collecting and analyzing the condition of coral reef ecosystems. *Ecosphere* , *8*(10). https://doi.org/10.1002/ecs2.1971

Flot, J.-F., Magalon, H., Cruaud, C., Couloux, A., & Tillier, S. (2008). Patterns of genetic structure among Hawaiian corals of the genus Pocillopora yield clusters of individuals that are compatible with morphology. *Comptes Rendus Biologies*, *331*(3), 239–247.

Flot, J.-F., & Tillier, S. (2007). The mitochondrial genome of Pocillopora (Cnidaria: Scleractinia) contains two variable regions: the putative D-loop and a novel ORF of unknown function. *Gene*, *401*(1-2), 80–87.

Fox, J., Weisberg, S., Adler, D., Bates, D., Baud-Bovy, G., Ellison, S., Firth, D., Friendly, M., Gorjanc, G., & Graves, S. (2012). Package "car." *Vienna: R Foundation for Statistical Computing*.

Jackson, A. L., Inger, R., Parnell, A. C., & Bearhop, S. (2011). Comparing isotopic niche widths among and within communities: SIBER - Stable Isotope Bayesian Ellipses in R. *The Journal of Animal Ecology*, *80*(3), 595–602.

LaJeunesse, T. C., Loh, W. K. W., van Woesik, R., Hoegh-Guldberg, O., Schmidt, G. W., & Fitt, W. K. (2003). Low symbiont diversity in southern Great Barrier Reef corals, relative to those of the Caribbean. *Limnology and Oceanography*, *48*(5), 2046–2054.

LaJeunesse, T. C., & Thornhill, D. J. (2011). Improved resolution of reef-coral endosymbiont

(Symbiodinium) species diversity, ecology, and evolution through psbA non-coding region genotyping. *PloS One*, *6*(12), e29013.

Lenth, R., Singmann, H., Love, J., Buerkner, P., & Herve, M. (2018). Emmeans: Estimated marginal means, aka least-squares means. *R Package Version*, *1*(1), 3.

Oksanen, J., Blanchet, F. G., Kindt, R., Legendre, P., Minchin, P. R., O'hara, R. B., Simpson, G. L., Solymos, P., Stevens, M. H. H., & Wagner, H. (2013). Package "vegan." *Community Ecology Package, Version*, *2*(9), 1–295.

R Core Team. (2021). *R: A language and environment for statistical computing*. https://www.R-project.org/

Sampayo, E. M., Dove, S., & LaJeunesse, T. C. (2009). Cohesive molecular genetic data delineate species diversity in the dinoflagellate genus Symbiodinium. *Molecular Ecology*. https://onlinelibrary.wiley.com/doi/abs/10.1111/j.1365-294X.2008.04037.x

Schmidt-Roach, S., Lundgren, P., Miller, K. J., & Gerlach, G. (2013). Assessing hidden species diversity in the coral Pocillopora damicornis from Eastern Australia. *Coral Reefs* . https://link.springer.com/content/pdf/10.1007/s00338-012-0959-z.pdf

Schmidt-Roach, S., Miller, K. J., & Lundgren, P. (2014). With eyes wide open: a revision of species within and closely related to the Pocillopora damicornis species complex (Scleractinia; Pocilloporidae) using morphology …. *Zoological Journal of the Linnean Society*. https://academic.oup.com/zoolinnean/article-abstract/170/1/1/2433469

Siebeck, U. E., Marshall, N. J., Klüter, A., & Hoegh-Guldberg, O. (2006). Monitoring coral bleaching using a colour reference card. In *Coral Reefs* (Vol. 25, Issue 3, pp. 453–460). https://doi.org/10.1007/s00338-006-0123-8

Swofford, D. L., & Others. (2002). *Phylogenetic analysis using parsimony (* and other methods)*. Version.

Turnham, K. E., Wham, D. C., Sampayo, E., & LaJeunesse, T. C. (2021). Mutualistic microalgae co-diversify with reef corals that acquire symbionts during egg development. *The ISME*

Journal, *15*(11), 3271–3285.

Wickham, H. (2016). *ggplot2: elegant graphics for data analysis*. Springer.
